# Pasta-Making Quality QTLome From Mediterranean Durum Wheat Landraces

**DOI:** 10.3389/fpls.2018.01512

**Published:** 2018-10-16

**Authors:** Martina Roselló, Conxita Royo, Fanny Álvaro, Dolors Villegas, Ruyman Nazco, Jose Miguel Soriano

**Affiliations:** Sustainable Field Crops Programme, Institute for Food and Agricultural Research and Technology, Lleida, Spain

**Keywords:** association mapping, grain quality, protein content, gluten strength, test weight, yellow color, sedimentation index

## Abstract

In order to identify genome regions related to pasta-making quality traits, association mapping (AM) was performed in a set of 165 durum wheat landraces from 21 Mediterranean countries. The collection was genotyped using 1149 DArT markers and 872 of them with a known genetic position were used for AM. The collection was grown in north-east Spain during 3 years. Results of ANOVA showed that trait variation for quality traits, except for grain protein content (GPC), was mainly explained by genetic effects. Landraces showed higher GPC than modern cultivars but lower gluten strength (GS). Modern and eastern landraces showed the highest yellow color index (YI). Balkan landraces showed the lowest test weight (TW). A total of 92 marker-trait associations were detected, 20 corresponding to GS, 21 to GPC, 21 to YI and 30 to TW. With the aim of detecting new genomic regions involved in grain quality, the position of the associations was compared with previously mapped QTL by a meta-QTL analysis. A total of 249 QTLs were projected onto the same map used for AM, identifying 45 meta-QTL (MQTL) regions and the remaining 15 QTLs as singletons. The position of known genes involved in grain quality was also included, and gene annotation within the most significant regions detected by AM was carried out using the wheat genome sequence.

## Introduction

Durum wheat (*Triticum turgidum* L. var. *durum*) is a staple food for 40% of the world's population (Peng J. et al., 2011), and the Mediterranean Basin is the largest production area in the world (Nazco et al., 2012). Flour and semolina are used to make many traditional Mediterranean foods, such as pasta, couscous, bulgur and flat bread, and pasta is the most common durum end product consumed in Europe, America and West Asia. For pasta making, durum wheat grains must possess specific traits related to flour quality. The most challenging objective of durum wheat breeding programmes should not be restricted to yield increases, as generally occurred during the twentieth century (Duveiller et al., 2007), but grain quality traits appropriate for pasta making should also be considered to meet social demands (Groos et al., 2007).

Grain protein quantity and quality are highly depending on the amount and type of glutenins and gliadins, proteins that are the main components of gluten and are responsible for the viscoelastic properties and extensibility of dough, respectively. Other important traits for pasta making are flour color and yellow pigment content, kernel weight, test weight and the starch characteristics related to grain hardness (Ruiz et al., 2005). Genetic improvement of durum wheat quality is subject to many constraints (Goutam et al., 2013). First, the quality traits are of a quantitative nature, and besides the known major genes determining gluten composition (glutenins and gliadins, reviewed in Ruiz et al., 2005), protein content (*Gpc-B1*, Olmos et al., 2003) and color (*Psy-A1*, Patil et al., 2008; *Psy-B1*, Elouafi et al., 2001), many other genes control their expression. Second, the quality traits require assessment of an end product, but direct estimation through milling and baking is costly and time-consuming, and requires a large grain sample. Indirect tests, such as the sodium dodecyl sulfate (SDS) sedimentation test, the alveograph and the mixograph, were developed to solve these problems but are still time-consuming. Alternative tools need to be sought, and the use of molecular markers can help identify new sources of desirable genes through association mapping (AM) and accelerated breeding programmes using marker-assisted selection (MAS). Association mapping is a complementary approach to traditional bi-parental linkage mapping. It uses linkage disequilibrium to identify genotype-phenotype associations, providing broader allelic variation and higher mapping resolution (Flint-Garcia et al., 2003; Breseghello and Sorrells, 2006). Because of the quantitative nature of grain quality traits, MAS will not replace traditional wheat quality testing procedures for the screening of advanced generations and cultivar evaluation. However, it will be an extremely valuable tool that allows breeders to identify new lines of interest for a more in-depth analysis at an earlier stage in the breeding programmes (Goutam et al., 2013).

Durum wheat originated in the Fertile Crescent approximately 10000 BP and spread across the Mediterranean Basin (Feldman, 2001). During the migration, natural and human selection resulted in the establishment of local landraces adapted to the prevalent climatic conditions (Peng J. H. et al., 2011). Due to their wide genetic diversity, landraces are considered useful for breeding as a source of new alleles (Lopes et al., 2015).

Grain protein content and composition are the main determinants of the end-use value of durum wheats. For that reason, in wheat breeding programs it is important to develop wheat cultivars with well-balanced grain protein compositions. Advances in grain quality resulting from breeding activities conducted during the twentieth century resulted in a loss of genetic variability that may constrain breeding for quality in the future (Nazco et al., 2014a; Subirà et al., 2014). This is the case of LMW glutenin subunits, which are restricted to a few alleles in modern cultivars. The large allelic variability found in Mediterranean landraces in previous studies (Moragues et al., 2006; Nazco et al., 2014a) provides tools for enhancing and diversifying gluten characteristics. Landraces may also provide new allelic sources for improving grain yellowness (Campos et al., 2016), and for improving grain weight due to the large genetic component of the trait (Nazco et al., 2012; Soriano et al., 2016). However, past breeding activities reduced grain protein content (Nazco et al., 2012; Subirà et al., 2014), possibly as a consequence of the negative relationship between this trait and yield. Therefore, exploring genetic diversity within pre-breeding materials is of interest to identify sources that allow protein content to be increased without a yield penalty.

The main objective of this study was to identify genome regions related to pasta-making quality traits through association mapping using a set of 165 Mediterranean durum wheat landraces representative of the genetic background existing in the species within the Mediterranean Basin. Additional goals were (i) to conduct a meta-analysis to restrict the QTL intervals and discover consensus QTL regions affecting the target traits, and (ii) to ascertain whether a geographic structure exists for the identified QTLs.

## Materials and methods

### Plant material

The germplasm used consisted of a collection of 172 durum wheat landraces and old cultivars from 21 Mediterranean countries and 20 modern cultivars (Supplementary Material 1).

Seeds were provided by public gene banks (CRF-INIA, Spain, ICARDA germplasm bank and USDA germplasm bank) and were purified and increased in bulk, as described by Nazco et al. (2012). The term “old cultivars” was used to designate a limited number of entries (6) corresponding to some of the first varieties obtained from selections within landraces (i.e., Andalucia 344 and Aziziah 17/45) or by crosses with landraces (i.e., Carlo jucci, Trinakria, Hymera, and Lozen 76). Given that old cultivars did not cluster apart from landraces according to the structure results of Soriano et al. (2016) both landraces and old cultivars were designated as landraces onwards. The collection was divided into five genetic subpopulations (SPs), one of the modern cultivars and four of landraces corresponding to their geographical origin: the eastern Mediterranean (EM, 19 cultivars), the eastern Balkans and Turkey (EB+T, 21 cultivars), the western Balkans and Egypt (WB+E, 33 cultivars), and the western Mediterranean (WM, 73 cultivars). Finally, 19 cultivars were classified as admixed. Due to missing genetic and phenotypic data, only 165 landraces and 18 modern varieties were analyzed.

### Phenotyping

Field experiments were carried out during three harvesting seasons (2007, 2008, and 2009) in Lleida, north-east Spain, under rain-fed conditions following a non-replicated modified augmented design with three replicated checks (the cultivars “Claudio,” “Simeto,” and “Vitron”) with plots of 6 m^2^ (8 rows, 5 m long with 0.15-m spacing). Sowing density was adjusted to 250 viable seeds m^−2^.

The plots were mechanically harvested at commercial maturity and a sample of 250 g of harvested grain from each plot was cleaned and used for quality tests. The analyzed traits were grain protein content (GPC), gluten strength (GS), yellow color index (YI) and test weight (TW). Additionally, sedimentation index (SI) was calculated as the quotient between GS and GPC and was expressed as mL/protein unit. GPC (%) was determined by a near-infrared spectroscope (NIT, Infratec® 1241 grain analyser, Foss Tecator AB, Sweden) calibrated against the standard Kjeldahl method (Kjeldahl, 1883). Whole-grain flour samples were obtained with a whole-meal grinder; fine particle size was ensured by attaching a 0.5-mm screen to the grinder. GS (mL) was determined on 1 g of flour samples by the SDS sedimentation test using the method of Axford et al. (1978), as modified by Peña et al. (1990). The YI (b, CIE L^*^a^*^b color system) was estimated on whole-grain flour by a portable reflectance colorimeter (CR-400, Konica-Minolta Sensing, Inc., Tokyo) equipped with a filter tri-stimulate system. Yellow pigment content (YPC) was measured following the AACC method, as described in Santra et al. (2003). TW (kg/hL) was determined by the GAC2100 analyser (Dickey-John Co., Auburn, IL, USA). Subsequently, the EU quality index (QI) for durum (European Commission Regulation No 2237/2003, 23 Dec, 2003; Official Journal of 24.12.2003; Royo and Briceño-Félix, 2011) was calculated from these four quality traits using the cultivars “Simeto,” “Gallareta,” and “Vitron” as reference checks and weighting each trait according to the following percentages: GPC (40%), GS (30%), YI (20%), and TW (10%).

### Statistical analysis

Phenotypic data were fitted to a linear mixed model with the check cultivars as fixed effects and the row number, column number and cultivar as random effects (Littell et al., 1997). Restricted maximum likelihood was used to estimate the variance components and to produce the best linear unbiased predictors (BLUPs) for the quality traits of each cultivar and year with the SAS-STAT statistical package (SAS Institute Inc, Cary, NC, USA). Combined ANOVAs were performed across experiments through the GLM procedure of the SAS-STAT statistical package (SAS Institute Inc, Cary, NC, USA), considering the cultivar and the year as random effects, and the year × cultivar interaction as the error term. The sum of squares of the cultivar effect was partitioned into differences between SPs and differences within them. Means were compared using the Tukey test (Tukey, 1949) with the JMP v12Pro statistical package (SAS Institute Inc, Cary, NC, USA). ANOVAs and mean comparisons were carried out using only the structured cultivars (146 landraces and 18 modern cultivars).

### Genotyping

DNA isolation was performed following the method of Doyle and Doyle (1987) from young leaf samples and sent to Diversity Arrays Technology Pty Ltd (Canberra, Australia) (http://www.diversityarrays.com). Genotyping was carried out using the durum wheat PstI/TaqI array v2.0. A total of 1149 DArT markers were used to genotype the whole collection and were ordered according with the consensus map of durum wheat developed by Maccaferri et al. (2014). Markers with duplicated patterns, with more than 20% of values missing and with minor allele frequency lower than 5% were excluded from the analysis.

### Association mapping

Association mapping was performed for the BLUPs of the measured traits in all the landraces for each year and across years using a mixed linear model (MLM) at the optimum compression level, accounting for the genetic relatedness and population structure (K+Q model) determined in Soriano et al. (2017). The mapping was performed using TASSEL software version 5.0 (Bradbury et al., 2007). The threshold *p*-value for considering a marker-trait association (MTA) significant was defined for each year and trait based on the Q-Q plot pattern at the point at which the observed *F*-test statistics deviated from the expected *F*-test statistics (Supplementary Material 2), as described in Sukumaran et al. (2012).

### QTL meta-analysis

Twenty published studies were examined and reported a total of 345 QTLs related to GPC, GS, YI, YPC and TW. For all of the studies, the following information was collected: parents of the cross, type of cross, number of progenies, name of QTLs, trait, environment, LOD score, phenotypic variance explained (PVE) by each QTL, QTL position on the author's linkage map, flanking markers and QTL confidence interval (CI). To compare the QTLs detected in different populations, original QTL data were projected onto the consensus map of durum wheat developed by Maccaferri et al. (2014). QTLs were projected following the homothetic approach proposed by Chardon et al. (2004). CIs were defined as reported by Soriano and Royo (2015) and estimated at 95% on the consensus map using the empirical formula proposed by Darvasi and Soller (1997) and Guo et al. (2006):

$$\begin{array}{l} \text{CI}=163/\left(\text{N}\times {\text{R}}^{2}\right)\text{for recombinant inbred line}\left(\text{RIL}\right)\\ \text{CI}=530/\left(\text{N}\times {\text{R}}^{2}\right)\text{for doubled haploid}\left(\text{DH}\right),\text{backcrosses}\left(\text{BC}\right)\\ \text{and}{\text{F}}_{2}\text{progenies} \end{array}$$

where *N* is the size of the population and *R*^2^ the proportion of variance explained by the QTL.

QTL meta-analysis was conducted using BioMercator v4.2 (Sosnowski et al., 2012) following the approach of Goffinet and Gerber (2000) when the number of QTLs in a chromosome was lower than 10 and that of Veyrieras et al. (2007) when the number of QTLs was 10 or more.

Additionally, genes involved in grain quality previously mapped or reviewed (Elouafi et al., 2001; Olmos et al., 2003; Ruiz et al., 2005; Pozniak et al., 2007; Patil et al., 2008, 2009; Zhang and Dubcovsky, 2008; Maccaferri et al., 2014) were also projected onto the consensus map (Maccaferri et al., 2014) for further comparisons.

Graphical representation of the genetic position of the significant MTAs, MQTLs and quality trait genes was carried out using MapChart 2.3 (Voorrips, 2002).

### Gene annotation

Gene annotation for the target region of the most significant MTAs was performed using the gene models for high-confidence genes reported for the wheat genome sequence (IWGSC RefSeq v1.0), available at https://wheat-urgi.versailles.inra.fr/Seq-Repository/. Intervals were defined by a genetic distance of 1cM above and below the corresponding marker or the linkage disequilibrium block (identified by TASSEL version 5.0 software) when present. Correspondence between genetic and physical distances was performed individually for each marker using the position of common markers in the consensus map (Maccaferri et al., 2014) and the wheat genome sequence.

## Results

### Phenotypic data

**Figure 1:**
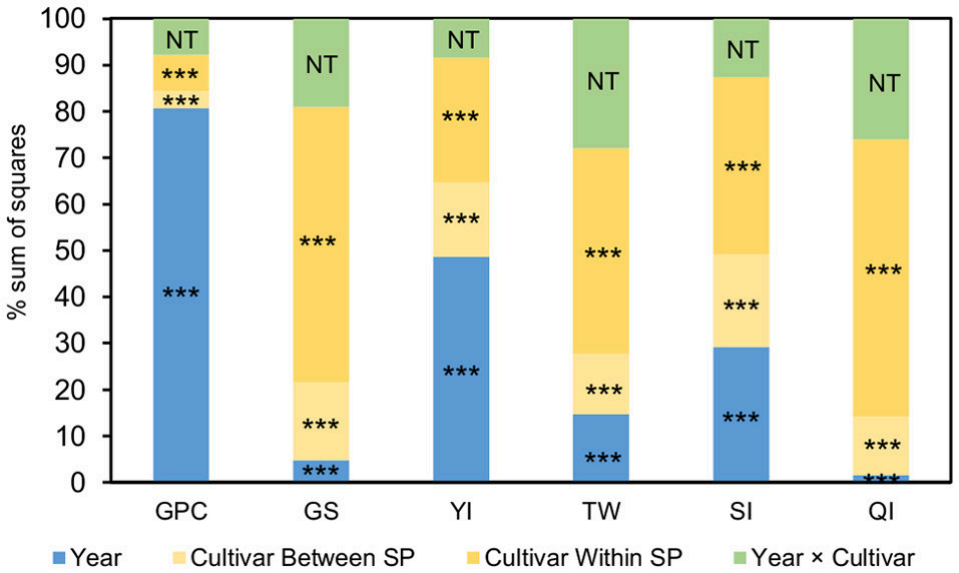
Percentage of the sum of squares (SS) of the ANOVA model for the pasta-making quality traits measured in 3 years on a collection of 183 durum wheat cultivars clustered in 5 genetic subpopulations (SPs). The SS of the genotype effect is partitioned into cultivar differences between SPs and cultivar differences within SPs. ^***^P < 0.001. GPC, grain protein content; GS, gluten strength; YI, yellow color index; TW, test weight; SI, sedimentation index; QI, quality index; NT, not testable.

The results of the ANOVA showed that variation in GPC was mostly explained by the environmental conditions of the year, whereas the remaining traits were influenced by large genetic effects mainly due to variations within SP (Figure 1).

**Figure 2:**
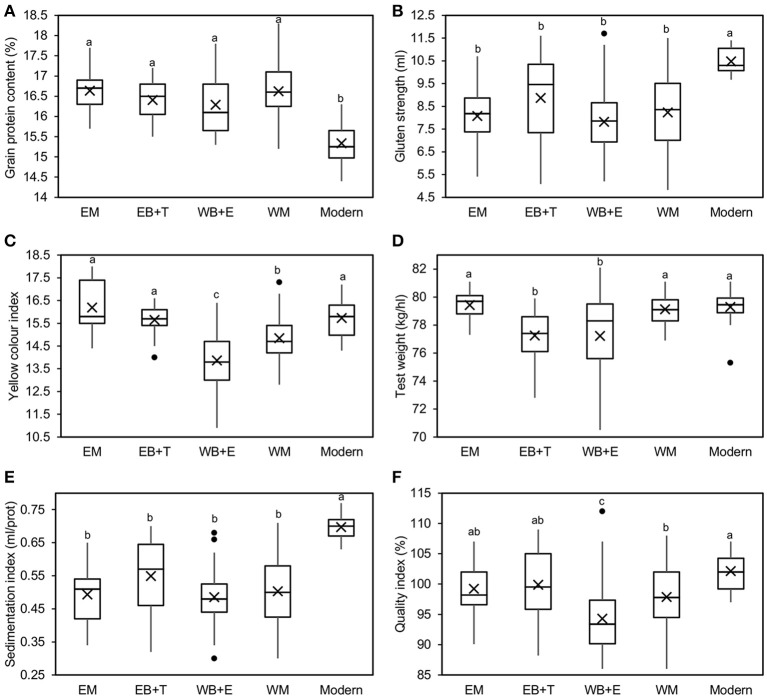
Boxplots for mean comparison of pasta-making quality traits [**(A)** grain protein content, **(B)** gluten strength, **(C)** yellow color index, **(D)** test weight, **(E)** sedimentation index and **(F)** quality index] in a collection of 183 durum wheat cultivars divided into five genetic SPs and grown for 3 years. EM, eastern Mediterranean; EB+T, eastern Balkans and Turkey; WB+E, western Balkans and Egypt; WM, western Mediterranean. Means within boxplots with different letters are significantly different at *P* < 0.05 following a Tukey test. × represents the mean value and ∙ represents outlier values.

Mean comparisons between SPs showed significant differences between modern cultivars and landraces in terms of GPC and GS (Figure 2). Regardless of their geographical origin, landraces had a higher GPC but a lower GS and SI than modern cultivars. For YI, cultivars from the WB+E had the lowest value (13.9), whereas cultivars from the EM showed the highest (16.2), but showed no statistically significant differences in comparison with modern cultivars (15.7) and the SPs from the EB+T (15.6). Balkan SPs showed lower TW values than landraces from the east and west of the Mediterranean Basin and modern cultivars. Finally, comparison of means for QI indicated that modern cultivars had the highest overall quality (102%), though it was similar to that of the SPs from the EM and the EB+T, while landraces from the WB+E showed the lowest quality (94%). This SP, however, showed the greatest variability for most traits, with some individuals showing the largest overall quality: e.g., the cultivar “D-2” from Egypt had the highest QI (112%) and GS (11.7 mL) and the cultivar “Heraldo del Rhin” had the highest TW (82.1 kg/hL).

### Association mapping

The landrace collection was genotyped using 1149 DArT markers. In order to reduce the risk of false positive MTAs, markers and cultivars were analyzed for the presence of duplicated patterns and missing values. Markers and cultivars were excluded as follows: 46 markers with duplicated patterns, 5 markers with more than 20% of values missing, and 24 markers with allele frequency lower than 5%. A total of 1074 markers remained. DArT markers were ordered according with the consensus map for durum wheat developed by Maccaferri et al. (2014), and only 872 with a known map position were used for mapping purposes. According to Soriano et al. (2017) using the same set of markers linkage disequilibrium decay was estimated up to 8 cM.

**Figure 3:**
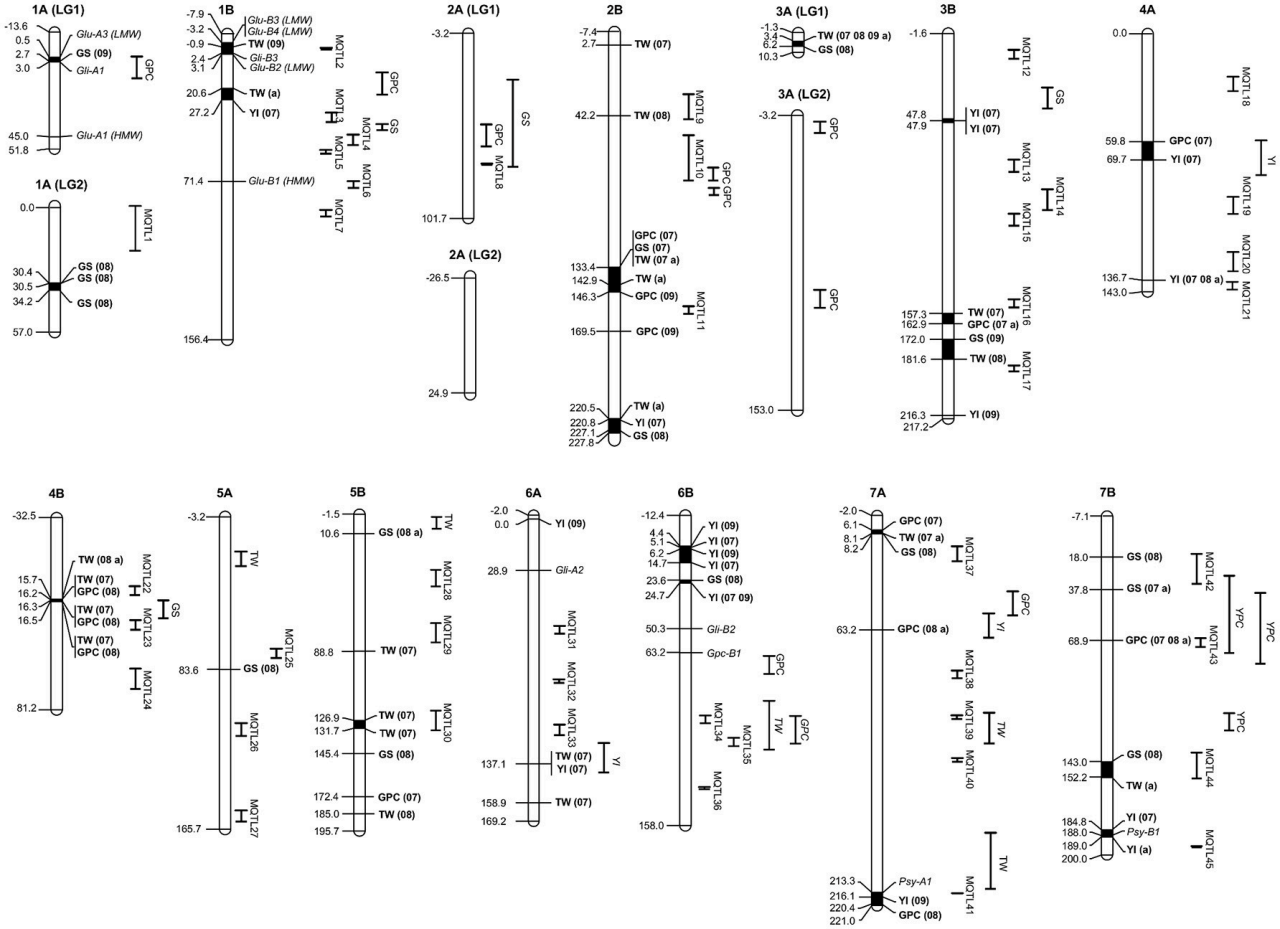
Genetic position of significant MTAs in the durum wheat consensus map developed by Maccaferri et al. (2014), together with the position of MQTLs reported in this study and known genes affecting grain quality. MTAs and their traits are indicated in bold. Numbers in parentheses after a trait represent the number of environments with significant MTAs. Single QTLs are reported as the trait for that QTL. 07, MTA significant in 2007; 08, MTA significant in 2008; 09, MTA significant in 2009; a, MTA significant across years. GPC, grain protein content; GS, gluten strength; YI, yellow color index; YPC, yellow pigment content; TW, test weight.

The results of the association mapping are reported in Table 1 and Figure 3. A total of 92 MTAs involving 70 markers were identified. A significance threshold for each trait and year was established considering the deviation of the observed from the expected test statistics in the Q-Q plots (Sukumaran et al., 2012), in all cases –log(*P*) > 2.0 (Supplementary Material 2). The MTAs were located in 13 chromosomes, with 36 and 64% of the MTAs located on genomes A and B respectively. The highest number of MTAs (12) were identified on chromosomes 2B and 7A, whereas on chromosome 5A only one was reported. Twenty MTAs corresponded to GS (including 18 markers), 21 to GPC and YI (15 and 18 markers, respectively) and 30 to TW (24 markers) (Table 1). Eighteen MTAs were found significant across years, four of them, wPt-2737 (GPC) and wPt-1140, wPt-1441 and wPt-6204 (TW), overcoming a *P* < 0.001 threshold (Supplementary Material 3). Twelve markers were involved in two or more MTAs (5 for GPC, 2 for GS, 1 for YI and 4 for TW). The marker wPt-2737 on chromosome 7B was detected for GPC with a –log(*P*) > 3.0 in 2 years and across years, explaining the highest percentage of PVE for the trait in this last association. Three other markers located in the same position on chromosome 7A (wPt-3883, wPt-7734 and wPt-9796) showed associations in 2008 and across the 3 years, and finally the marker wPt-2698 (3B) was detected in 2007 and across years. For GS, rPt-6127 and wPt-7653 were associated in at least 1 year and across years. For YI the marker wPt-1437 showed associations for 2007 and 2009 (showing the highest PVE for the trait in this year) and wPt-3729 showed three associations (2007, 2008 and across years). For TW, the marker wPt-6204 showed associations for the 3 years and across years, with a –log(*P*) > 3, wPt-1140 and wPt-1441 each showed two associations with TW (2007 and across years), and the association between wPt-1140 and 2007 was the MTA with the highest PVE for TW. Finally, the marker wPt-8892 was detected in 2008 and across years, and the marker wPt-1140 was also associated with GPC and GS in 2007.

**Table 1:** Significant markers associated with pasta-making quality traits obtained in 165 Mediterranean durum wheat landraces.

**Trait**	**Marker**	**Year**	**Chromosome**	**Position (cM)**	**-log(*P*)**	**Marker *R*^2^**
GPC (%)	wPt-1140	2007	2B	133.4	3.63	0.09
	wPt-8693	2009	2B	146.3	2.73	0.07
	wPt-4223	2009	2B	169.5	2.59	0.06
	wPt-2698	2007	3B	162.9	2.32	0.05
	wPt-2698	Across years	3B	162.9	2.39	0.05
	wPt-7355	2007	4A	59.8	3.00	0.08
	wPt-6123	2008	4B	16.2	2.56	0.06
	wPt-5497	2008	4B	16.3	2.56	0.06
	tPt-5342	2008	4B	16.5	3.22	0.08
	wPt-7400	2007	5B	172.4	2.34	0.05
	wPt-6959	2007	7A	6.1	2.74	0.06
	wPt-3883	2008	7A	63.2	2.61	0.06
	wPt-3883	Across years	7A	63.2	2.76	0.06
	wPt-7734	2008	7A	63.2	2.64	0.06
	wPt-7734	Across years	7A	63.2	2.84	0.07
	wPt-9796	2008	7A	63.2	2.64	0.06
	wPt-9796	Across years	7A	63.2	2.77	0.06
	wPt-4220	2008	7A	220.4	2.19	0.05
	wPt-2737	2007	7B	68.9	3.12	0.08
	wPt-2737	2008	7B	68.9	3.34	0.08
	wPt-2737	Across years	7B	68.9	4.30	0.11
GS (ml)	wPt-6280	2009	1A.1	2.7	3.48	0.08
	wPt-1310	2008	1A.2	30.4	2.88	0.07
	wPt-6853	2008	1A.2	30.4	2.84	0.07
	wPt-1011	2008	1A.2	30.5	2.87	0.07
	wPt-5274	2008	1A.2	34.2	2.53	0.07
	wPt-6754	2008	1A.2	34.2	2.00	0.05
	wPt-1140	2007	2B	133.4	2.94	0.07
	wPt-6894	2008	2B	227.1	2.50	0.06
	wPt-6854	2008	3A.1	6.2	2.11	0.05
	wPt-11691	2009	3B	172	2.95	0.07
	tPt-0353	2008	5A	83.6	2.03	0.04
	rPt-6127	2008	5B	10.6	2.56	0.06
	rPt-6127	Across years	5B	10.6	2.61	0.06
	wPt-6880	2008	5B	145.4	2.49	0.06
	wPt-7954	2008	6B	23.6	2.06	0.05
	wPt-2056	2008	7A	8.2	2.11	0.05
	wPt-1853	2008	7B	18	3.07	0.08
	wPt-7653	2007	7B	37.8	2.73	0.06
	wPt-7653	Across years	7B	37.8	2.79	0.06
	wPt-4258	2008	7B	143	2.89	0.08
YI	wPt-2694	2007	1B	27.2	2.48	0.06
	wPt-2724	2007	2B	220.8	2.18	0.04
	wPt-8140	2007	3B	47.8	2.42	0.05
	wPt-1349	2007	3B	47.9	2.92	0.07
	wPt-8686	2007	3B	47.9	3.04	0.07
	wPt-2416	2009	3B	216.3	2.42	0.05
	wPt-0162	2007	4A	69.7	2.25	0.05
	wPt-3729	2007	4A	136.7	2.20	0.05
	wPt-3729	2008	4A	136.7	2.70	0.06
	wPt-3729	Across years	4A	136.7	2.61	0.06
	wPt-8443	2009	6A	0	2.39	0.05
	wPt-3247	2007	6A	137.1	2.32	0.05
	wPt-3774	2009	6B	4.4	2.60	0.06
	wPt-0245	2007	6B	5.1	2.56	0.06
	wPt-7662	2009	6B	6.2	2.76	0.06
	wPt-5673	2007	6B	14.7	2.45	0.05
	wPt-1437	2007	6B	24.7	2.52	0.06
	wPt-1437	2009	6B	24.7	3.22	0.08
	wPt-1429	2009	7A	216.1	2.85	0.06
	wPt-5228	2007	7B	184.8	2.24	0.05
	wPt-5138	Across years	7B	189	2.68	0.06
TW (Kg/hl)	wPt-2654	2008	1B	−0.9	2.84	0.06
	wPt-5562	Across years	1B	20.6	2.19	0.04
	wPt-1634	2007	2B	2.7	2.38	0.04
	wPt-7158	2008	2B	42.2	2.66	0.06
	wPt-1140	2007	2B	133.4	4.51	0.10
	wPt-1140	Across years	2B	133.4	3.60	0.08
	wPt-8569	Across years	2B	142.9	2.40	0.06
	wPt-7360	Across years	2B	220.5	2.17	0.04
	wPt-6204	2007	3A.1	3.4	3.45	0.07
	wPt-6204	2008	3A.1	3.4	3.41	0.08
	wPt-6204	2009	3A.1	3.4	3.14	0.07
	wPt-6204	Across years	3A.1	3.4	4.01	0.09
	wPt-8480	2007	3B	157.3	2.13	0.04
	wPt-2491	2008	3B	181.6	2.35	0.05
	wPt-8892	2007	4B	15.7	3.51	0.07
	wPt-8892	Across years	4B	15.7	2.53	0.05
	wPt-6123	2007	4B	16.2	2.26	0.04
	wPt-5497	2007	4B	16.3	2.26	0.04
	tPt-5342	2007	4B	16.5	2.16	0.04
	wPt-6022	2007	5B	88.8	2.43	0.05
	wPt-2707	2007	5B	126.9	3.68	0.08
	wPt-6191	2007	5B	126.9	3.60	0.07
	wPt-4577	2007	5B	131.7	3.70	0.08
	tPt-3714	2008	5B	185	2.55	0.06
	wPt-9000	2007	6A	137.1	2.39	0.05
	wPt-6995	2007	6A	158.9	2.20	0.04
	wPt-1441	2007	7A	8.1	3.01	0.06
	wPt-1441	Across years	7A	8.1	3.38	0.07
	wPt-5343	Across years	7B	152.2	2.54	0.05
	wPt-8615	Across years	7B	152.2	2.36	0.05

*GPC, grain protein content; GS, gluten strength; YI, yellow color index; TW, test weight*.

According to the results of Soriano et al. (2017) reporting a LD decay up to 8 cM depending on the chromosome and the approach used by Laidò et al. (2014), MTAs located within a region of 5–10 cM are considered as belonging to the same QTL. Thus, the 92 MTAs were restricted to 37 QTLs (named as MTA-QTLs) (Table 2). The number of MTAs per QTL ranged from 1 in 14 MTA-QTLs to 8 in 1 MTA-QTL. Taking into account the number of traits involved in each MTA-QTL, 65% of the MTA-QTLs involved only 1 trait, 27% involved 2 traits and the remaining 8% 3 traits. Twenty-three out of the 37 MTA-QTLs had 2 or more MTAs. Of those, 5 MTA-QTLs were detected in one environment, 10 in 2 environments, 7 in 3 environments, and finally 1 in four environments.

**Table 2:** MTA-QTLs.

**MTA-QTL**	**Traits involved**	**MTAs**	Environments^*^	**Chromosome**	**Region (cM)**
mtaq1A.1	GS	1	1	1A	2.7
mtaq1A.2	GS	5	1	1A	30.4–34.2
mtaq1B.1	TW	1	1	1B	0.0
mtaq1B.2	TW, YI	2	2	1B	20.6–27.2
mtaq2B.1	TW	1	1	2B	2.7
mtaq2B.2	TW	1	1	2B	42.2
mtaq2B.3	GPC, GS,TW	6	3	2B	133.4–146.3
mtaq2B.4	GPC	1	1	2B	169.5
mtaq2B.5	GS, TW, YI	3	3	2B	220.5–227.1
mtaq3A.1	GS, TW	5	4	3A	3.4–6.2
mtaq3B.1	YI	3	1	3B	47.8–47.9
mtaq3B.2	GPC, TW	3	2	3B	157.3–162.9
mtaq3B.3	GS, TW	2	2	3B	172–181.6
mtaq3B.4	YI	1	1	3B	216.3
mtaq4A.1	GPC, YI	2	1	4A	59.8–69.7
mtaq4A.2	YI	3	3	4A	136.7
mtaq4B.1	GPC, TW	8	3	4B	15.7–16.5
mtaq5A.1	GS	1	1	5A	83.6
mtaq5B.1	GS	2	2	5B	10.6
mtaq5B.2	TW	1	1	5B	88.8
mtaq5B.3	TW	3	1	5B	126.9–131.7
mtaq5B.4	GS	1	1	5B	145.4
mtaq5B.5	GPC	1	1	5B	172.4
mtaq5B.6	TW	1	1	5B	185.0
mtaq6A.1	YI	1	1	6A	0.0
mtaq6A.2	TW, YI	2	1	6A	137.1
mtaq6A.3	TW	1	1	6A	158.9
mtaq6B.1	YI	4	2	6B	4.4–14.7
mtaq6B.2	GS, YI	3	3	6B	23.6–24.7
mtaq7A.1	GPC, GS, TW	4	3	7A	6.1–8.2
mtaq7A.2	GPC	6	2	7A	63.2
mtaq7A.3	GPC, YI	2	2	7A	216.1–220.4
mtaq7B.1	GS	1	1	7B	18.0
mtaq7B.2	GS	2	2	7B	37.8
mtaq7B.3	GPC	3	3	7B	68.9
mtaq7B.4	GS, TW	3	2	7B	143–152.2
mtaq7B.5	YI	2	2	7B	184.8–189.0

*GPC, grain protein content; GS, gluten strength; YI, yellow color index; TW, test weight*.

* *Number of environments represent the number of years and/or across years where an MTA was significant*.

The analysis of the distribution among SPs of the significant markers for GPC across years showed that wPt-2737 was mainly present in the EM SPs, in contrast with the EB+T and modern SPs, which lacked this allele with a positive effect in most cultivars (Table 3). Protein content increased significantly on average by 3.6% in cultivars holding wPt-2737 (Table 3). Markers wPt-3883, wPt-7734 and wPt-9796 on chromosome 7A were present in most of the cultivars of each SP (from 70% in modern and WB+E cultivars to 100% in WM and EB+T cultivars), increasing GPC on average by 5.0%. Finally, wPt-2698 was mainly present in landraces (from 80% in WM cultivars to 100% in eastern cultivars) but showed a negative effect, decreasing protein content by 1%. The marker was present in 60% of the modern cultivars. The frequency of the markers showing a positive effect in the upper 10th percentile ranged from 0.8 (wPt-2737) to 1 (wPt-3883, wPt-7734 and wPt-9796), while in the lower 10th percentile the frequency of the marker wPt-2737 dropped to 0.055. Considering the extreme landraces, the five with the highest GPC (“Morocco,” “D-2,” “Sinai no.8,” “Dezassete,” and “IG-95841”) carried the four markers, whereas in the ones with the lowest GPC (“Haj Mouline,” “Ruso,” “VII/18-X24,” “37,” and “1P1”), only marker wPt-2737 was missing.

**Table 3:** (A) Frequency of cultivars within each SP holding markers with significant associations with each trait at least in one year and across years and (B) mean values for each pasta-making quality trait of cultivars holding or not holding each marker.

		**Grain protein content (%)**					**Gluten strength (mL)**		**Yellow index**	**Test weight (kg/hL)**			
	***N***	**wPt-2737**	**wPt-3883**	**wPt-7734**	**wPt-9796**	**wPt-2698**	**rPt-6127**	**wPt-7653**	**wPt-3729**	**wPt-6204**	**wPt-1140**	**wPt-1441**	**wPt-8892**
**A**													
EastMediterranean	19	0.7	0.9	0.8	1.0	1.0	0.9	0.3	0.8	1.0	1.0	0.8	1.0
East Balkans - Turkey	21	0.1	1.0	1.0	1.0	1.0	0.9	0.2	0.9	0.5	1.0	0.4	1.0
West Balkans - Egypt	33	0.4	0.7	0.7	0.7	0.9	0.6	0.4	0.2	0.9	0.5	0.3	0.8
West Mediterranean	73	0.3	1.0	1.0	1.0	0.8	0.7	0.2	0.2	0.9	1.0	0.6	0.9
Modern	18	0.1	0.7	0.7	0.7	0.6	1.0	0.2	0.8	0.9	0.4	0.3	0.6
**B**													
Absence		16.2 b	15.6 b	15.6 b	15.6 b	16.5 a	7.31 b	8.31 a	14.5 b	75.9 b	79.3 a	78.7 a	77.7 a
Presence		16.8 a	16.5 a	16.5 a	16.5 a	16.4 a	8.74 a	8.83 a	15.7 a	78.8 a	78.4 b	78.4 a	78.6 a

*Means within columns with different letters are significantly different at P < 0.05*.

Marker wPt-7653, associated with GS, was present in 40% of the WB+E landraces and had a similar frequency (20–30%) to the remaining SPs. On the other hand, marker rPt-6127, whose presence significantly increased GS by 16.4% on average, was widely distributed across the Mediterranean Basin, being present in all modern cultivars. This marker was identified in all landraces of the upper 10th percentile and only in 44% of genotypes in the lowest 10th percentile. Taking into account only landraces, the five genotypes with the highest GS (“D-2,” “BGE019265,” “Trigo Glutinoso,” “5P4,” and “Vroulos”) carried the marker, whereas the five with the lowest GS (“Razza 181,” “Akathiotico Naurotheri,” “VII/13-X11,” “IG-96851,” and “Blanco de Corella”) lacked it.

For YI, wPt-3729 was mainly restricted to the eastern regions and most modern cultivars. The marker was present in 83% of the upper 10th percentile genotypes and only in 5% of the genotypes of the lower 10th percentile. Additionally it was present in the five landraces with highest YI values (“Harani Auttma,” “Safra Maan,” “26,” “Hati,” and “Safra Jerash”) and absent in the five landraces with the lowest values (“28,” “441-IX/97,” “Heraldo del Rhin,” “440-IX/96,” and “Dalmatia 3”).

Markers associated with TW showed a different distribution among SPs. The marker wPt-6240, with the strongest effect on TW (3.6%), was present in the majority of cultivars of all SPs, but in the EB+T the frequency decreased to 50%. It was also present in almost 90% of modern cultivars. The marker was present in all landraces of the upper 10th percentile and in the five with the highest TW (“Heraldo del Rhin,” “Espanhol,” “Harani Auttma,” “Haiti,” and “Rubio de Montijo”), but it was also present in 61% of the genotypes included in the lower 10th percentile, and in three out of five landraces with the lowest TW (“5P4,” “28 Giza,” “D-2,” and “BGE019265”). Markers wPt-1140 and wPt-1441 were associated with low TW. The first marker was present in all cultivars from the EM, EB+T and WM SPs, but its frequency was lower in the WB+E and modern cultivars. It was present in most genotypes of both 10th percentiles, 89% for the upper and 94% for the lower. Moreover, it was present in four and five landraces showing the highest and lowest TW, respectively. The second marker (wPt-1441) was present mainly in the WM and EM SPs, decreasing in landraces from the Balkans and in modern cultivars. This marker was present only in 50% of the genotypes from the upper 10th percentile and in 67% of those from the lower 10th percentile. The marker was present only in one out of the five landraces with the highest TW and in the five landraces with the lowest TW. Finally, marker wPt-8892, with a low positive effect on TW, was present in all eastern cultivars and in a high percentage of western cultivars, but its presence decreased to 60% in modern cultivars. It was present in a high percentage in both 10th percentiles (94 and 78% for the upper and lower ones, respectively) and in four and three out of five landraces with the highest and lowest TW.

### QTL meta-analysis

In order to compare the genomic regions involved in grain quality identified by association mapping with previous reported QTLs, a QTL meta-analysis was carried out. The analysis collected data from 345 QTLs in 20 studies published from 2003 to 2016 reporting QTLs for GPC (129), GS (79), YI (37), YPC (74) and TW (26) (Supplementary Material 4). The study covered 21 experimental crosses in 93 different environments. Of the 345 QTLs, 249 were subjected for projection onto the consensus map developed by Maccaferri et al. (2014). The remaining QTLs were not suitable for projection due to the lack of common markers between original and consensus maps.

Of the 249 projected QTLs, 43% were found in genome A and 57% in genome B. QTLs were detected in all chromosomes, ranging from 2 QTLs in chromosome 3A to 42 QTLs in chromosome 1B. The PVE by single QTLs followed an L-shaped distribution, with the majority of QTLs showing a low PVE (< 0.20 for 82%) (Supplementary Material 5). Phenotypic variance explained a range from 0.01 to 0.542 with an average of 0.14. Size of the CIs ranged from 1.4 to 175.3 cM with an average of 18.0 cM. Approximately two-thirds of the QTLs (64%) had CIs < 20 cM (Supplementary Material 5).

The 249 QTLs projected onto the consensus map (Maccaferri et al., 2014) were subjected to meta-analysis. Following an Akaike information criterion, 204 QTLs were grouped into 45 meta-QTLs (MQTLs) (Table 4). The software did not include in the analysis 10 QTLs with low LOD scores and/or large CIs. Twenty QTLs were excluded as their CIs overlapped with different MQTLs, and it was not possible to determine the MQTL to which they belonged based on the membership coefficient given by the software. Fifteen QTLs remained as single QTLs not overlapping with other QTLs or MQTLs. Figure 3 shows the position of the MQTLs and the single QTLs. The number of QTLs clustered in a single MQTL ranged from 2 to 20 (Table 4). The CI for the MQTLs ranged from 0.1 to 24.3 cM, with an average of 6.4 cM, indicating a significant reduction of 64% in the CIs from the initial QTLs. Seventeen MQTLs were related to a single trait.

**Table 4:** Summary of MQTL information for grain quality traits.

**Chromosome**	**MQTL**	**Position^a^**	**CI^b^**	**Left marker**	**Right marker**	**N QTL**	**Traits**
1A LG2	1	0.5	24.3	cfa2129	wPt-11558	2	YPC
1B	2	0.0	1.10	wPt-2654	wPt-8627	8	GS
1B	3	33.0	5.06	gwm18	ksum28	9	TW, GPC, YPC
1B	4	49.0	5.57	barc302	wmc419	7	GS, YPC, YI
1B	5	55.5	2.09	wmc419	wPt-9937	5	GS
1B	6	73.0	3.55	kbo-0425	wPt-0506	4	GS
1B	7	88.4	3.41	wPt-5011	rPt-7906	4	GPC, GS
2A LG1	8	71.0	0.81	gwm95	gwm372	11	GPC, YPC, YI
2B	9	32.0	4.86	wmc112	wPt-7932	5	TW, GPC
2B	10	57.1	6.93	gwm429	wPt-6477	2	GPC
2B	11	157.0	2.72	wPt-7305	wmc332	4	GPC
3B	12	10.4	5.09	cfb6018	gpw7774	4	TW, GPC
3B	13	73.6	6.94	CA499601b	wPt-10530	3	GPC
3B	14	92.9	11.8	wPt-5390	wmc1	3	TW, GS
3B	15	104.0	6.81	wPt-0446	barc164	2	GPC, GS
3B	16	152.0	4.70	wPt-7145	wPt-0384	5	GPC, YPC
3B	17	187.0	3.25	wPt-4401	wPt-6956	2	YI
4A	18	27.8	8.01	gwm610	Lp-A3	5	GPC, YPC
4A	19	95.1	9.42	wPt-1584	wPt-1701	2	GPC, YI
4A	20	126.0	10.6	wPt-3596	wPt-7354	3	GS, YI
4A	21	140.0	4.32	wmc723	wPt-9103	6	TW, GPC, YPC, YI
4B	22	10.6	5.26	gwm857	gwm368	2	TW, GPC
4B	23	31.0	5.92	wPt-1491	gwm781	2	GPC, GS
4B	24	62.7	12.1	wPt-8092	wPt-9625	2	GPC
5A	25	70.6	4.94	barc197	gwm639	3	GPC
5A	26	112.0	6.97	wPt-730410	barc142	3	GS
5A	27	159.0	5.89	gwm126	gwm291	2	TW
5B	28	38.4	10.3	wPt-1951	gwm213	2	GPC
5B	29	72.3	12.0	wmc415	kbo-0077	3	GPC, YPC, YI
5B	30	127.0	12.2	wPt-6014	barc140	2	GPC
6A	31	62.0	4.46	gwm132	gwm4675	4	YPC, YI
6A	32	90.7	2.03	gwm1150	wPt-2014	18	GS, YPC, YI
6A	33	118.0	5.59	gwm169	BE483091	2	YPC, YI
6B	34	100.0	4.22	wPt-6889	R18-370	2	TW, GPC
6B	35	112.0	4.55	gwm1682	850	2	GPC
6B	36	137.0	1.23	barc125a	wPt-5270	7	GS
7A	37	19.8	8.65	gwm1187	wmc168	3	GS
7A	38	88.7	4.38	wmc83	cfa2147	4	TW, GPC, YPC, YI
7A	39	113.0	2.11	gwm573	wPt-0321	2	GPC
7A	40	137.0	2.26	barc29	barc121	7	GS, YPC, YI
7A	41	214.0	0.11	BJ262177B	gwm1061	6	YPC, YI
7B	42	25.1	18.2	wmc323	wmc405	2	GPC, YI
7B	43	70.1	5.36	wPt-8273	gwm540a	6	TW, GPC, YPC, YI
7B	44	145.0	15.8	barc315	kbo-0372	2	GPC, YPC
7B	45	195.0	0.68	Psy-B1	wPt-7387	20	GPC, YPC, YI

*GPC, grain protein content; GS, gluten strength; YI, yellow color index; YPC, yellow pigment content; TW, test weight*.

a *Most probable position on the consensus map*.

b *Length of the 95% confidence interval (CI) centered on the most probable position in cM*.

In addition to the integration of reported QTLs, genes involved in grain quality previously mapped were also projected onto the consensus map (Maccaferri et al., 2014) (Table 5, Figure 3). Only one MTA for GS (wPt-6280 in 2009) was observed in the region of chromosome 1A, where the complex *Glu-A3/Gli-A1* has been mapped. However, no relation was observed between the presence of the marker and any of the 15 different alleles at this locus identified by Nazco et al. (2014a) in the same collection. No other MTA for GS of the 20 identified in this work mapped close to a locus with a known mapping position coding for HMW or LMW-GS. Three MTAs for YI were placed in the vicinity of the phytoene synthase genes *Psy-A1* (wPt-1429) and *Psy-1B* (wPt-5228 and wPt-5138).

**Table 5:** List of genes involved in grain quality previously mapped and projected onto the consensus map.

**Gene**	**Trait**	**References**
*Gpc-B1*	Protein content	Olmos et al., 2003
*Gli-A1*	Gluten strength	Patil et al., 2009
*Gli-A2*	Gluten strength	Ruiz et al., 2005; Maccaferri et al., 2014
*Gli-B2*	Gluten strength	Maccaferri et al., 2014
*Gli-B3*	Gluten strength	Ruiz et al., 2005; Patil et al., 2009
*Glu-A3*	Gluten strength	Ruiz et al., 2005; Patil et al., 2009
*Glu-B1*	Gluten strength	Patil et al., 2009; Maccaferri et al., 2014
*Glu-B2*	Gluten strength	Patil et al., 2009
*Glu-B3*	Gluten strength	Patil et al., 2009
*Psy-A1*	Yellow color	Patil et al., 2008
*Psy-B1*	Yellow color	Elouafi et al., 2001; Pozniak et al., 2007

### Gene annotation

Candidate genes within 1cM above and below the most significant markers identified by association mapping were detected using the high-confidence gene annotation from the wheat genome sequence (IWGSC RefSeq v1.0) (International Wheat Genome Sequencing Consortium, 2018). Using the position of common markers in the consensus map (Maccaferri et al., 2014) and the wheat genome sequence, genetic distances were converted to physical distances. Annotated genes for the corresponding regions are shown in Supplementary Material 6.

For GPC, two loci were selected: the locus comprising markers wPt-3883, wPt-7734, and wPt-9796 located at the same position (63.2 cM) on chromosome 7A, and wPt-2737 located at 68.9 cM on chromosome 7B. For the locus at chromosome 7A the correspondence was 1:1.5 (genetic:physical). The evaluated region of approximately 3.5 Mb showed 72 gene models. One gene located at 64.6 Mb was also included in the interval because of its homology with previously identified genes increasing GPC. For the locus on chromosome 7B the correspondence was 1:15. The physical region analyzed covered a distance of 30 Mb with 119 gene models.

For TW, markers wPt-1140 (133.4 cM, 2B) and wPt-6204 (3.4 cM, 3A) were analyzed. The region covered for the former was 4 Mb (ratio 1:2) and included 24 gene models. As marker wPt-6204 was not mapped on the wheat genome sequence and the sequence of the DArT clone was not available on the Diversity Arrays Technology website (www.diversityarrays.com) to perform BLAST, its position was defined by the SSR marker barc294, mapped by Maccaferri et al. (2014) in the same genetic position. The physical interval on the wheat genome sequence to this region corresponded to 4.6 Mb (ratio 1:2.3) and 94 gene models were found within it.

For YI only the marker wPt-3729 (136.7 cM, 4A) was selected, as the marker wPt-1437, which was also present in two MTAs, was not mapped on the wheat genome, and the sequence of the DArT clone was not available on the website to perform BLAST against the genome sequence. The marker was included in a linkage disequilibrium block with three other close markers, covering a genetic region of 0.1 cM. For this region, the ratio of genetic to physical distance was 1:2.5 and the region covering 5 Mb surrounding the marker had 101 gene models.

Finally, for GS, as occurred for YI, only one marker among others that were significant in at least 2 years or across years was identified in the genome sequence and was included in the analysis. The physical region for the marker wPt-7653 (38.7 cM, 7B) was identified through the linked marker wPt-7064 and the ratio of genetic to physical distance was defined as 1:2.3. Fifty-two gene models were included within the approximately 4.7 Mb flanking region.

## Discussion

### Quality traits

Results of ANOVA showed the variability existing in the phenotypic expression of pasta-making quality traits in durum wheat. Cultivar effect was partitioned into variation within and between the genetic SPs defined by Soriano et al. (2016). GPC was largely explained by the environmental effect (harvesting year), whereas for GS, TW, SI and QI the cultivar accounted for a larger variation than environment, suggesting a higher genetic control for these traits. These results agree with those of previous studies reporting a large environmental influence on durum wheat GPC in Mediterranean environments (Rharrabti et al., 2001, 2003; De Vita et al., 2007; Taghouti et al., 2010). For YI, although the environment effect was higher, the genotypic effect was also large (48 and 43% of the explained variance, respectively). When the genotype effect was partitioned, most of the variation accounted for all studied traits within SPs, revealing an enormous intra-population variability. These results based on classification of genetic SPs (Soriano et al., 2016) are in agreement with those reported by Nazco et al. (2012) using a population structure based on the different climatic zones of the Mediterranean Basin.

When comparing mean differences among SPs, modern cultivars showed the highest values for overall quality and sedimentation indices, probably due to the higher GS of the cultivars belonging to this SP. In agreement with these results, a previous study that analyzed the changes caused by breeding in quality traits of Italian and Spanish durum wheat reported a lack of progress in TW, a loss of GPC and a substantial improvement in GS and yellow color (Subirà et al., 2014). The high level of GPC found in Mediterranean landraces in this and previous studies (Nazco et al., 2012) was associated in the current study with a high frequency of markers with a positive and significant effect on GPC, thus offering a potential tool for protein content improvement in breeding programmes.

A previous study that used the same germplasm as the present one demonstrated that the greater GS values found in modern cultivars were due to a very few allelic combinations of high (HMW-) and low (LMW-) molecular weight glutenin subunit loci (Nazco et al., 2014a), which could be a constraint for future quality improvement. However, allelic banding patterns drastically increasing GS were identified in landraces (Nazco et al., 2014b), showing their potential to broaden the genetic basis for gluten quality improvement.

Significant differences in YI appeared between two groups: the highests values were found in modern cultivars and landraces from EM countries, whereas the lowest ones were found in western SPs. These results suggest that yellow color of wheat grain decreased during the migration of wheat from the Fertile Crescent, the area of origin and domestication, to the west of the Mediterranean Basin, and was recently improved by breeding, as demonstrated with the use of historical series of genotypes (Subirà et al., 2014).

For TW we found a large genetic component accounting for the total variance explained (57%) and only 14% due to environment. These results disagree with those reported by Taghouti et al. (2010) and Subirà et al. (2014), who found a large environmental effect accounting for the variation of the trait. A possible explanation for these differences may be the lower number of genotypes used by those authors compared with the large collection used in the current study. The largest differences between SPs appeared between landraces from the Balkans peninsula and the remaining SPs, the latter showing heavier grains but much lower internal variability.

### Genetic architecture

In addition to the studies conducted by Nazco et al. (2014a,b) that used glutenin subunit composition to study the genetic bases of GS, this study is one of the first attempts to elucidate the molecular bases of pasta-making quality traits in Mediterranean durum wheat landraces. The collection of landraces was grown under the dry and warm conditions typical of the Mediterranean Basin (Royo et al., 2014). A genome-wide association study (GWAS) was performed following a mixed linear model method accounting for the genetic relatedness between cultivars and their population structure (K+Q model) in order to reduce the number of spurious associations.

A total of 92 associations involving 4 traits and 70 markers were detected in 3 years and across years in north-eastern Spain. As reported previously by Laidò et al. (2014), MTAs located within short map intervals (ca. 5–10 cM) should be considered as belonging to the same QTL. Thus, following this suggestion in the present study, 37 genomic regions (or quality MTA-QTLs) involving the 92 MTAs were identified. Eight of these regions were detected across 3 and 4 environments and were considered the most stable QTLs. MTAs were widely distributed across the genome, with all chromosomes except 2A showing significant associations.

In order to compare the MTA-QTLs identified in the present research with previously reported QTLs, a QTL meta-analysis was carried out, summarizing data from 249 QTLs for quality traits published from 2003 to 2016. These QTLs were projected onto the same consensus map (Maccaferri et al., 2014). The meta-analysis revealed the presence of 60 genomic regions (45 MQTLs + 15 singletons) controlling quality traits in the genomes A and B of wheat. The meta-analysis produced a simplification in the genome regions containing QTLs, as their number was reduced up to four times and the CI also diminished significantly by 64%. Eleven out of the 37 MTA-QTLs reported in the present study were located within the CI of MQTLs and five of them had QTLs for the same traits. On chromosome 2B, the *mtaq2B.2* for TW was located within the interval of MQTL9, which had QTLs for TW and GPC. On chromosome 4A, *mtaq4A.1*, which had MTAs for GPC and YI, was located with a single QTL for YI reported by Roncallo et al. (2012). Additionally, in the same chromosome, the *mtaq4A.2* associated with YI was flanked closely by two MQTLs (20 and 21), both of them with QTLs for YPC and YI also described by Roncallo et al. (2012). The importance of this region on chromosome 4A for flour yellow color lies in the stability across years found in the present study. Additionally, Roncallo et al. (2012) found epistatic effects of these QTLs on chromosome 4A, with others located on the bottom of chromosomes 6A and 7A, where MTAs for YI were also found in the present work: *mtaq6A.2*, which was located within the CI of a QTL for YI; and *mtaq7A.3*, also with MTAs for GPC, which mapped with MQTL41, which had QTLs for GPC, YI and YPC. Finally, the *mtaq7B.3* for GPC was identified in the region of MQTL43, which had six QTLs for GPC, TW, YI and YPC. The MTA for GPC in this region was also considered stable, as it was detected in two years and across years. Although other genomic regions containing MTAs were located within MQTL positions, they identified QTLs for different traits to those reported by association analysis. Thus, most MTA-QTLs identified by association analysis corresponded to new regions for quality traits present in durum wheat Mediterranean landraces.

Previous studies reported the association of molecular markers with grain quality traits. Laidò et al. (2014) performed a GWAS for different agronomical, morphological and grain quality traits using a collection of 230 inbred lines, 128 of them corresponding to durum wheat cultivars and 102 to wild and domesticated cultivars from six other subspecies. These authors found 39 MTAs for GPC in the whole collection, only 14 of them corresponding to the durum wheat cultivars. Only one MTA from the durum cultivars corresponded to an MTA-QTL located in the present study (*mtaq3B.3*) harboring associations with GS and TW. When the whole collection was analyzed, 3 MTAs were placed in a similar location to *mtaq4A.1, mtaq7A.1*, and *mtaq7A.2*, all of them associated with GPC. More recently, Giraldo et al. (2016) reported an association analysis for agro-morphological and grain quality traits in a structured collection of Spanish durum wheat landraces including different subspecies (*T. durum, T. turgidum*, and *T. diccocon*). The authors found 33 MTAs for quality traits (2 for GPC, 26 for GS, 3 for TW and 2 for YI) and 6 of them can be integrated within MTA-QTLs reported in the current study: wPt-8780 for GS in *mtaq1A.1* (GS), on chromosome 3B; wPt-3599 (TW) in *mtaq3B.2* (GPC, TW); wPt-0990 (GS) in *mtaq3B.3* (GS, TW); wPt-0665 (YI) in *mtaq3B.4* (YI); wPt-6916 (GS) in *mtaq6B.2* (GS, YI); and finally, wPt-6869 (YI) in *mtaq7B.5* (YI). Interestingly, all common associations between the study of Giraldo et al. (2016) and ours corresponded to the same quality traits. The low number of common MTAs detected between the current study and those reported by Laidò et al. (2014) and Giraldo et al. (2016) could be explained by the different plant material used by the three groups: durum wheat inbred lines (Laidò et al., 2014) and Spanish durum wheat landraces (Giraldo et al., 2016), both authors including other durum subspecies; and durum wheat landraces from 21 Mediterranean countries in our work. The findings of the present study highlight the importance of the Mediterranean landraces as a source of new alleles to improve durum wheat quality traits, as reported previously by Nazco et al. (2012, 2014a,b). Another reason for the differences could be the different maps used for the GWAS. Laidò et al. (2014) and Giraldo et al. (2016) used the map reported by Marone et al. (2012) to locate the polymorphic markers, whereas the present study used the consensus map reported by Maccaferri et al. (2014).

Finally, to support the association of the MTA-QTLs with previously reported genes involved in pasta-making quality traits, known genes were also included in the map (Table 5). On chromosome 1A, the LMW glutenin locus *Glu-A3* (Ruiz et al., 2005) and the gliadin locus *Gli-A1* (Patil et al., 2009) were located in the vicinity of an MTA for GS, within *mtaq1A.1*. Additionally, a QTL for GPC was also located in this region (Suprayogi et al., 2009). On chromosome 2B the HMW and LMW glutenin loci *Glu-B2* and *Glu-B3* (Patil et al., 2009) and the gliadin *Gli-B3* (Ruiz et al., 2005; Patil et al., 2009) were located together with an MTA for TW in *mtaq1B.1* and with MQTL2, harboring 8 QTLs for GS. In the same chromosome HMW-GS *Glu-B1* (Patil et al., 2009; Maccaferri et al., 2014) was located within MQTL6, harboring 4 QTLs for GS. The *Gli-A2* locus identified by Ruiz et al. (2005) and Maccaferri et al. (2014) on chromosome 6A and *Gli-B2* (Maccaferri et al., 2014) on chromosome 6B were located in regions without MTAs or MQTLs. For protein content, the *GPC-1B* locus was located on chromosome 6B (Olmos et al., 2003) and was projected onto the consensus map close to a QTL for GPC (Prasad et al., 2003). Finally, for yellow color the phytoene synthase genes *Psy-A1* (Patil et al., 2008) and *Psy-1B* (Elouafi et al., 2001; Pozniak et al., 2007) were located within the YI MTA-QTLs *mtaq7A.3* and *mtaq7B.5*, respectively. In both cases, MQTLs for YI were also identified in the same regions (MQTL41 and MQTL45, respectively).

### Gene annotation

Potential candidate genes for the studied traits were searched using the high-confidence gene annotation from the wheat genome sequence (IWGSC RefSeq v1.0). The position of common markers between the durum wheat consensus map (Maccaferri et al., 2014) and the wheat genome sequence at https://wheat-urgi.versailles.inra.fr/Seq-Repository/ was used to define CIs. The limitation of using only DArT markers to find regions in the genome sequence reporting candidate genes resides in the uncovered regions for this type of markers in the sequence, as reported in this work for some of the MTAs. In this case closely linked markers or blasting the marker sequence (if available) resulted in useful approaches to identify gene models. If no closely linked markers or the marker sequence is not available the identification of candidate genes becomes a difficult task and highly saturated maps are needed. The join analysis of GWAS together with QTL meta-analysis using reference maps also helps to identify genome regions uncovered by DArT markers.

Among the gene models within CIs of 1 cM above and below the selected marker, candidate genes previously described in the literature were found for GPC and TW.

For GPC two regions were subjected to analysis. The first region on chromosome 7A comprised the markers wPt-3883, wPt-7734, and wPt-9796 located at 61.8 Mb, and the second one on chromosome 7B the marker wPt-2737 at 199.3 Mb. In both cases, the gene models TraesCS7A01G106300.1 (7A) and TraesCS7B01G143900.1 (7B) encoded for an NAC domain containing protein transcription factor. This kind of domain was described by Uauy et al. (2006) for *Gpc-B1* and is associated with an increase in GPC, Zn and Fe content in wheat. This transcription factor accelerates senescence and increases the nutrient remobilization from leaves to developing grains.

For TW the analyzed loci were wPt-1140 (601.9 Mb, 2B) and wPt-6204 (7.9 Mb, 3A). For wPt-1140, the gene model TraesCS2B01G419400.1 was found, encoding for an E3 ubiquitin protein ligase. This is the same kind of protein encoded by the gene *TaGW2-A1* (Simmonds et al., 2016), which has a role as a negative regulator of grain size and weight in hexaploid wheat (Yang et al., 2012). A mutant allele of this gene significantly increased grain weight, grain width and grain length in tetraploid and hexaploid wheat. For wPt-6204, several candidate genes within the defined CI were found. The gene model TraesCS3A01G007600.1 encodes for a transcription elongation factor as the gene *TaTEF-7A* (Zheng et al., 2014), which increases potential grain yield and yield-related traits and confers complex pleiotropic effects on growth, yield and quality. Two other gene models within the region of wPt-6204 were associated with expansin proteins (TraesCS3A01G011100.1 and TraesCS3A01G011200.1). According to Lizana et al. (2010), the expression of expansins in wheat is associated with grain size. The results of these authors support an association between the expression levels of expansins and fast growth of the wheat grain taking place at early developmental stages.

### Breeding potential

As a consequence of domestication and breeding, the genetic variability of crops has been gradually reduced. Exploiting genetic diversity from local landraces in breeding programmes is a valuable approach to recovering and to broadening allelic variation of traits of interest (Lopes et al., 2015). Mediterranean durum wheat landraces are an important group of genetic resources because of their specific adaptation to local environments and their end-product quality (Nazco et al., 2012), in view of the enormous genetic diversity found among Mediterranean landraces in traits of commercial importance (Soriano et al., 2016).

The results reported in the present study can be exploited to improve wheat cultivars by selecting the most significant and stable MTAs across environments. Protein content has decreased as a consequence of past breeding activities, which concentrated on increasing yield potential (Motzo et al., 2004; De Vita et al., 2007; Royo et al., 2008; Subirà et al., 2014). Marker wPt-2737 located on chromosome 7B shows the greatest variance explained for this trait; it is linked to an increase in GPC and is present mainly in EM cultivars. Further studies are needed in order to develop new molecular markers from the sequence of wPt-2737 and validate them in progenies and breeding material. As reported previously by Subirà et al. (2014), the greatest improvements for GS were produced with the introduction and release of the first improved cultivars in Italy and Spain, and were due to the use of a limited number of HMW- and LMW-GS alleles associated with GS. Thus, breeding for GS should focus on increasing the genetic diversity of glutenin allelic combinations rather than increasing the trait itself. In the current study, mean values for GS were higher in modern cultivars than in landraces, and the frequency of the markers with higher *R*^2^ and stability across environments did not differ between the two types of cultivar. The marker wPt-3729 for YI showed that eastern landraces and modern cultivars differed clearly from western landraces. Although the values of YI in modern cultivars is high, the use of eastern landraces to improve yellowness would help increase genetic diversity for the trait. Finally, for TW the maker wPt-6204 appeared to be the most stable across environments, as it was detected in all 3 years and across years. However, except for the EB+T SP, the marker was present in most cultivars belonging to the other landrace SPs and modern cultivars. Although markers wPt-1140 and wPt-1441 were mainly present in landraces, they produced a negative effect. The marker wPt-8892 would be the most suitable for increasing the trait in modern cultivars because it is present mainly in landraces.

Recently the genome sequence of the cultivar “Chinese Spring” was published (IWGSC 2018), becoming a useful tool for the wheat breeding community. The sequence will allow the identification of candidate genes through map based approaches, the development of new molecular markers in order to saturate genome regions or identifying new ones in low recombination regions, cloning candidate genes in other cultivars to study mutations and differences in expression levels. Knowing the whole sequence of candidate genes will also help in speeding breeding programs by the use of gene editing technologies.

## Author contributions

CR and JS obtained funding. CR, FA, DV, and JS designed the experiments. CR and DV assembled and purified the germplasm collection. DV and RN phenotyped the collection for quality traits. MR performed association mapping, meta-analysis and statistical analysis. FA and JS conceived the manuscript. MR, FA, and JS wrote the manuscript. CR, FA, and JS edited and provided a critical review of the manuscript. MR, CR, FA, DV, RN, and JS read and approved the final manuscript.

### Conflict of interest statement

The authors declare that the research was conducted in the absence of any commercial or financial relationships that could be construed as a potential conflict of interest.
