# Supplementary material for: Pasta-Making Quality QTLome From Mediterranean Durum Wheat Landraces
Source: Front Plant Sci. 2018 Oct 16;9:1512. doi: 10.3389/fpls.2018.01512 (PMC6232839; doi:10.3389/fpls.2018.01512)
Supplement: Supplementary file 1 [file Table_1.DOCX]

Supplementary Material

Pasta-making quality QTLome from Mediterranean durum wheat landraces

**Martina Roselló, Conxita Royo, Fanny Álvaro, Dolors Villegas, Ruyman Nazco, Jose Miguel Soriano***

*** Correspondence:**Jose Miguel Soriano
josemiguel.soriano@irta.cat

**Supplementary material 1.** Cultivars included in the study.

| Cultivar | Country | Genetic Subpopulation |
| --- | --- | --- |
| IG-83920 | Italy | East Mediterranean |
| IG-95812 | Syria | East Mediterranean |
| Hymera | Italy | East Mediterranean |
| Aziziah 17/45 | Italy | East Mediterranean |
| 9923 | Lebanon | East Mediterranean |
| 9929 | Lebanon | East Mediterranean |
| 9935 | Lebanon | East Mediterranean |
| Safra Jerash | Jordan | East Mediterranean |
| Hourah | Lebanon | East Mediterranean |
| Harani Auttma | Jordan | East Mediterranean |
| Horani Howawi | Jordan | East Mediterranean |
| Zugbieh Sutra | Jordan | East Mediterranean |
| Zoghbiyeh Safra | Jordan | East Mediterranean |
| Etith | Israel | East Mediterranean |
| Safra Maan | Jordan | East Mediterranean |
| 26 | Jordan | East Mediterranean |
| Hati | Israel | East Mediterranean |
| Tripshiro | Libya | East Mediterranean |
| Sinai No.8 | Egypt | East Mediterranean |
| Vroulos | Cyprus | East Balkan and Turkey |
| IG-82549 | Cyprus | East Balkan and Turkey |
| BGE018192 | Turkey | East Balkan and Turkey |
| BGE018350 | Turkey | East Balkan and Turkey |
| BGE018351 | Turkey | East Balkan and Turkey |
| BGE018353 | Turkey | East Balkan and Turkey |
| BGE-018354 | Turkey | East Balkan and Turkey |
| BGE019262 | Turkey | East Balkan and Turkey |
| BGE019263 | Turkey | East Balkan and Turkey |
| BGE019264 | Turkey | East Balkan and Turkey |
| BGE019265 | Turkey | East Balkan and Turkey |
| BGE019266 | Turkey | East Balkan and Turkey |
| BGE019270 | Turkey | East Balkan and Turkey |
| Belgrade 9 | Serbia | East Balkan and Turkey |
| 248-VII/7 | Macedonia | East Balkan and Turkey |
| 259-VII/12 | Macedonia | East Balkan and Turkey |
| VII/13-X11 | Macedonia | East Balkan and Turkey |
| 196/71 | Macedonia | East Balkan and Turkey |
| 1575 | Serbia | East Balkan and Turkey |
| II/4 | Macedonia | East Balkan and Turkey |
| Mavraani | Greece | East Balkan and Turkey |
| Blanco de Corella | Spain | West Balkan and Egypt |
| Blanquillo | Spain | West Balkan and Egypt |
| Gros de Cerdaña | Spain | West Balkan and Egypt |
| Heraldo del Rhin | Spain | West Balkan and Egypt |
| Pisana cañihueca | Spain | West Balkan and Egypt |
| Blanquillón de Boñar | Spain | West Balkan and Egypt |
| Milagro | Egypt | West Balkan and Egypt |
| Dezassete | Portugal | West Balkan and Egypt |
| Durazio Rijo Glabro | Portugal | West Balkan and Egypt |
| Alentejo | Portugal | West Balkan and Egypt |
| Caxudo de sete espigas | Portugal | West Balkan and Egypt |
| Zoco Yebel Hebil | Morocco | West Balkan and Egypt |
| D-2 | Egypt | West Balkan and Egypt |
| Dalmatia 1 | Croatia | West Balkan and Egypt |
| Dalmatia 3 | Croatia | West Balkan and Egypt |
| 5P4 | Egypt | West Balkan and Egypt |
| 1P1 | Egypt | West Balkan and Egypt |
| 356-I/9 | Montenegro | West Balkan and Egypt |
| 440-IX/96 | Croatia | West Balkan and Egypt |
| 441-IX/97 | Croatia | West Balkan and Egypt |
| VII/18-X24 | Macedonia | West Balkan and Egypt |
| Giza 2 | Egypt | West Balkan and Egypt |
| 23 | Montenegro | West Balkan and Egypt |
| 33 | Montenegro | West Balkan and Egypt |
| 37 | Montenegro | West Balkan and Egypt |
| 42 | Montenegro | West Balkan and Egypt |
| 56 | Croatia | West Balkan and Egypt |
| 2751 | Egypt | West Balkan and Egypt |
| MG 26429 | Egypt | West Balkan and Egypt |
| 28 | Egypt | West Balkan and Egypt |
| 31 | Egypt | West Balkan and Egypt |
| Mishriki | Egypt | West Balkan and Egypt |
| Girgeh | Egypt | West Balkan and Egypt |
| Arisnegro de Tenerife | Spain | West Mediterranean |
| Basto Duro | Spain | West Mediterranean |
| Candeal de Salamanca | Spain | West Mediterranean |
| Colorado de Jerez | Spain | West Mediterranean |
| Enano de Andújar | Spain | West Mediterranean |
| Fartó | Spain | West Mediterranean |
| Pinet | Spain | West Mediterranean |
| Raspinegro Canario | Spain | West Mediterranean |
| Raspinegro de Alcalá Guadaira | Spain | West Mediterranean |
| Recio de Almería | Spain | West Mediterranean |
| Verdial | Spain | West Mediterranean |
| Tchirpan | Bulgaria | West Mediterranean |
| Lozen 76 | Bulgaria | West Mediterranean |
| Carlantino | Italy | West Mediterranean |
| Cicirelo | Italy | West Mediterranean |
| IG-83905 | Italy | West Mediterranean |
| IG-92895 | Algeria | West Mediterranean |
| IG-92967 | Algeria | West Mediterranean |
| IG-93030 | Algeria | West Mediterranean |
| IG-93621 | Algeria | West Mediterranean |
| IG-94009 | Algeria | West Mediterranean |
| IG-96802 | Crete | West Mediterranean |
| IG-96851 | Crete | West Mediterranean |
| Alonso | Spain | West Mediterranean |
| Andalucía 344 | Spain | West Mediterranean |
| Azulejo de Villa del Río | Spain | West Mediterranean |
| Blancal | Spain | West Mediterranean |
| Claro de Balazote | Spain | West Mediterranean |
| Entrelargo de Montijo | Spain | West Mediterranean |
| Farto cañifino | Spain | West Mediterranean |
| Rubio de Miajadas | Spain | West Mediterranean |
| Rubio de Montijo | Spain | West Mediterranean |
| Ruso | Spain | West Mediterranean |
| Semental | Spain | West Mediterranean |
| Recio de Cañete | Spain | West Mediterranean |
| Carlo jucci | Italy | West Mediterranean |
| Senatore Capelli | Italy | West Mediterranean |
| Trinakria | Italy | West Mediterranean |
| Razza 208 | Italy | West Mediterranean |
| Balilla Falso | Italy | West Mediterranean |
| Mindium | Turkey | West Mediterranean |
| Raposinho | Portugal | West Mediterranean |
| Reading | Egypt | West Mediterranean |
| Durazio Rijo | Portugal | West Mediterranean |
| Milazzo | Italy | West Mediterranean |
| Raspinegro | Portugal | West Mediterranean |
| Razza 181 | Italy | West Mediterranean |
| Anafil | Portugal | West Mediterranean |
| Espanhol | Portugal | West Mediterranean |
| Amarelo Barba Preta | Portugal | West Mediterranean |
| Tremes rijo | Portugal | West Mediterranean |
| Razza 96 | Italy | West Mediterranean |
| Dur de Medeah | Algeria | West Mediterranean |
| Maghoussa | Morocco | West Mediterranean |
| Merzaga | Morocco | West Mediterranean |
| Red Beard | Morocco | West Mediterranean |
| Souri | Tunisia | West Mediterranean |
| Realforte | Tunisia | West Mediterranean |
| Biskri | Tunisia | West Mediterranean |
| Morocco | Morocco | West Mediterranean |
| Saffi | Morocco | West Mediterranean |
| Beladi Rouge | France | West Mediterranean |
| Tounse | France | West Mediterranean |
| Trigo Glutinoso | France | West Mediterranean |
| Ble Dur 250 | Morocco | West Mediterranean |
| Oned Zenati | Morocco | West Mediterranean |
| Mahmoudi C | Morocco | West Mediterranean |
| Maghoussa Amizmiz | Morocco | West Mediterranean |
| Muri | Cyprus | West Mediterranean |
| Cobros | Morocco | West Mediterranean |
| Rapsani | Greece | West Mediterranean |
| Rubio enlargado d’Atlemteje | France | West Mediterranean |
| Reyati | Lebanon | West Mediterranean |
| IG-84856 | Lebanon | Admixed |
| IG-95841 | Syria | Admixed |
| IG-95931 | Syria | Admixed |
| Marques | Portugal | Admixed |
| Lobeiro de grao escuro | Portugal | Admixed |
| Abu Fashit | Israel | Admixed |
| Louri AP 5 | Tunisia | Admixed |
| De Santa Marta | France | Admixed |
| Iumillo | France | Admixed |
| 9918 | Lebanon | Admixed |
| Akathiotico Naurotheri | Cyprus | Admixed |
| Salti na Zinia | Jordan | Admixed |
| Juljulith | Israel | Admixed |
| FAO 29.912 | Cyprus | Admixed |
| Haj Mouline | Morocco | Admixed |
| JM-3987 | Israel | Admixed |
| JM-3989 | Israel | Admixed |
| 18/71 | Serbia | Admixed |
| Hamira | Tunisia | Admixed |
| Amilcar | Spain | Modern |
| Ancalei | Spain | Modern |
| Arment | France | Modern |
| Astigi | Spain | Modern |
| Boabdil | Spain | Modern |
| Bolido | Spain | Modern |
| Bolo | Spain | Modern |
| Claudio | Italy | Modern |
| Gallareta | CIMMYT | Modern |
| Hispasano | Spain | Modern |
| Jupare | CIMMYT | Modern |
| Kronos | USA | Modern |
| Meridiano | Italy | Modern |
| Ocotillo | USA | Modern |
| Simeto | Italy | Modern |
| Sula | CIMMYT | Modern |
| Svevo | Italy | Modern |
| Vitron | CIMMYT | Modern |

**Supplementary material 2.** Quantile-Quantile plot (QQ plot) of the observed F-test statistics versus the expected F-test statistics for the markers considered in the association mapping.


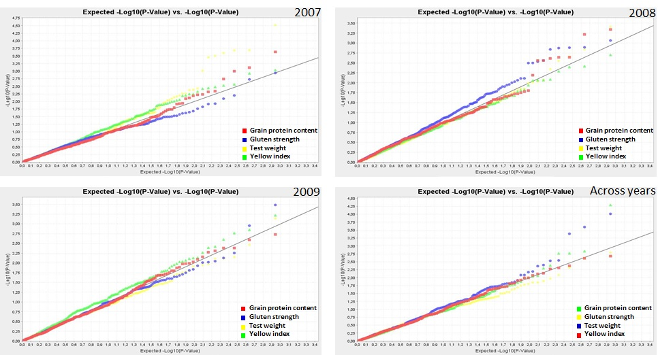


**Supplementary material 3.** Manhattan plots of the association mapping conducted with the mean values across years experiments for pasta-making quality traits. Chromosomes are depicted alternately in blue (A genome) and yellow (B genome). Thresholds from –log(*P*)>2.0 are indicated.


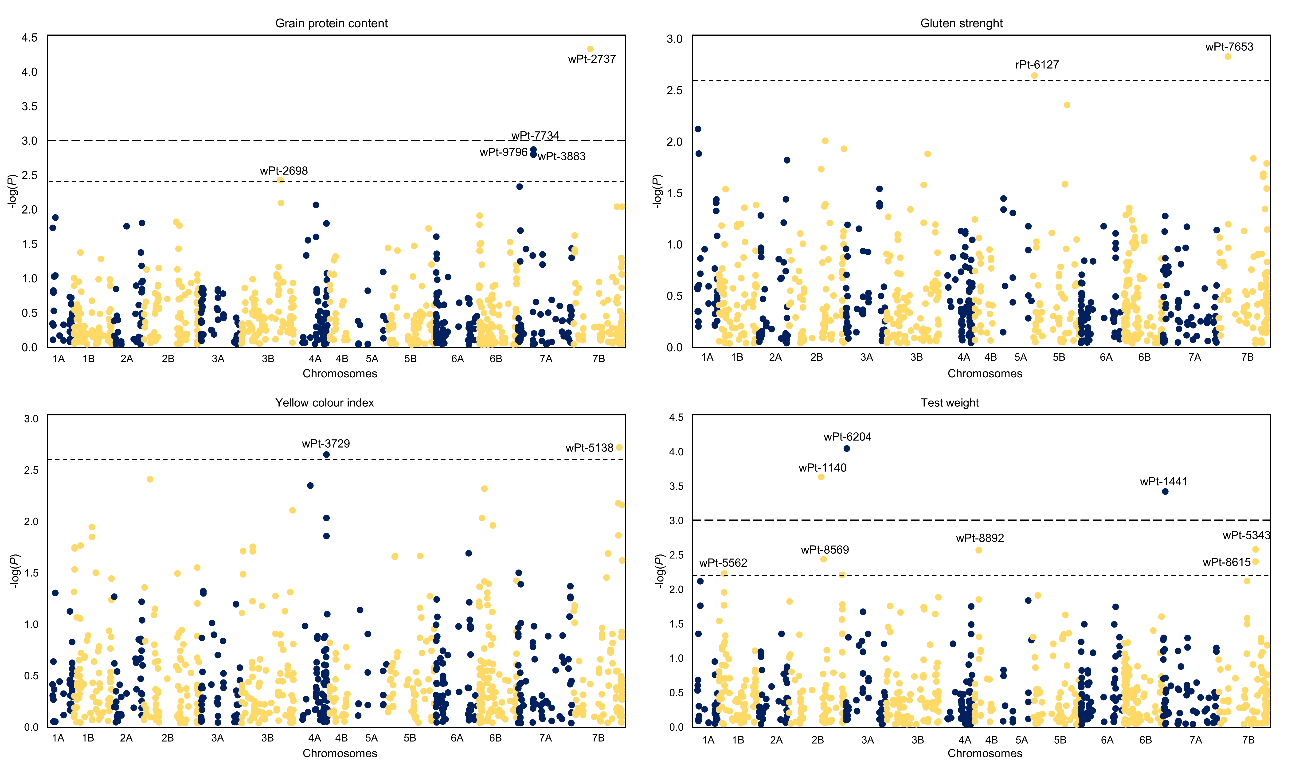


**Supplementary material 4.** Summary of the QTL studies reviewed in the meta-analysis. Number of QTLs for each trait in the studies are indicated.

| Reference | Cross | Population type | Size | Environments (n) | Grain protein content | Gluten strength | Yellow colour index | Yellow pigment content | Test weight |
| --- | --- | --- | --- | --- | --- | --- | --- | --- | --- |
| Blanco et al. 2011 | Latino x Primadur | F2:F3 | 121 | 4 | - | - | 10 | 20 | - |
| Conti et al. 2011 | UC1113 x Kofa | RIL | 93 | 6 | 15 | 26 | - | - | - |
| Groos et al. 2004 | Renan x Recital | RIL | 194 | 3 | 1 | 1 | - | - | - |
| Huang et al. 2006 | AC Karma x 87E03-S2B1 | DH | 414 | 4 | 1 | - | - | - | 3 |
| Kunert et al. 2007 | Batis x Syn022 | BC2F3:5 | 250 | 10 | 4 | - | - | - | 6 |
|  | Zentos x Syn086 | BC2F3:6 | 150 |  | 1 | - | - | - | 3 |
| Li et al. 2009 | Neixiang 188 x Yanzhan 1 | RIL | 198 | 2 | 14 | 13 | - | - | - |
| Ma et al. 2012 | Lang x CSCR6 | RIL | 82 | 4 | 2 | 1 | - | - | - |
| McCartney et al. 2006 | RL4452 x AC Domain | DH | 182 | 6 | 2 | 3 | - | - | - |
| Patil et al. 2008 | PDW 233 (YAV'S'/TEN'S') x Bhalegaon 4 | RIL F2:7 | 140 | 4 | - | - | - | 6 | - |
| Patil et al. 2009 | PDW 233 x Bhalegaon 4 | RIL | 140 | 4 | 1 | 11 | - | - | - |
| Prasad et al. 2003 | WL711 x PH132 | RIL | 100 | 5 | 8 | - | - | - | - |
| Roncallo et al. 2012 | UC1113 x Kofa | RIL | 93 | 6 | - | - | 27 | 24 | - |
| Sun et al. 2008 | Chuan 35050 x Shannong 483 | RIL | 131 | 2 | 3 | - | - | - | - |
| Sun et al. 2010 | Ning7840 x Clark | RIL | 132 | 7 | 2 | - | - | - | 6 |
| Sun et al. 2016 | Chuan 35050 × Shannong 483 | RIL | 131 | 6 | 7 | - | - | - | - |
| Suprayogi et al. 2009 | DT695 x Strongfield | DH | 185 | 6 | 19 | - | - | - | - |
| Tsilo et al. 2010 | MN98550 x MN99394 | RIL | 139 | 3 | 4 | - | - | - | - |
| Turner et al. 2004 | Avalon x Hobbit | RIL | 200 | 3 | 6 | - | - | - | - |
| Wang et al. 2012 | Weimai 8 × Luohan 2 | RIL | 302 | 3 | 28 | - | - | - | - |
| Zhang et al. 2008 | UC1113 x Kofa | RIL | 93 | 5 | 11 | 24 | - | 24 | 8 |
| Total |  |  |  | 93 | 129 | 79 | 37 | 74 | 26 |

RIL (recombinant inbred line); DH (double haploid); BC2 (backcross).

**Supplementary material 5.** Number of QTLs per: a) phenotypic variance explained and b) supporting interval, studied in the meta-analysis.


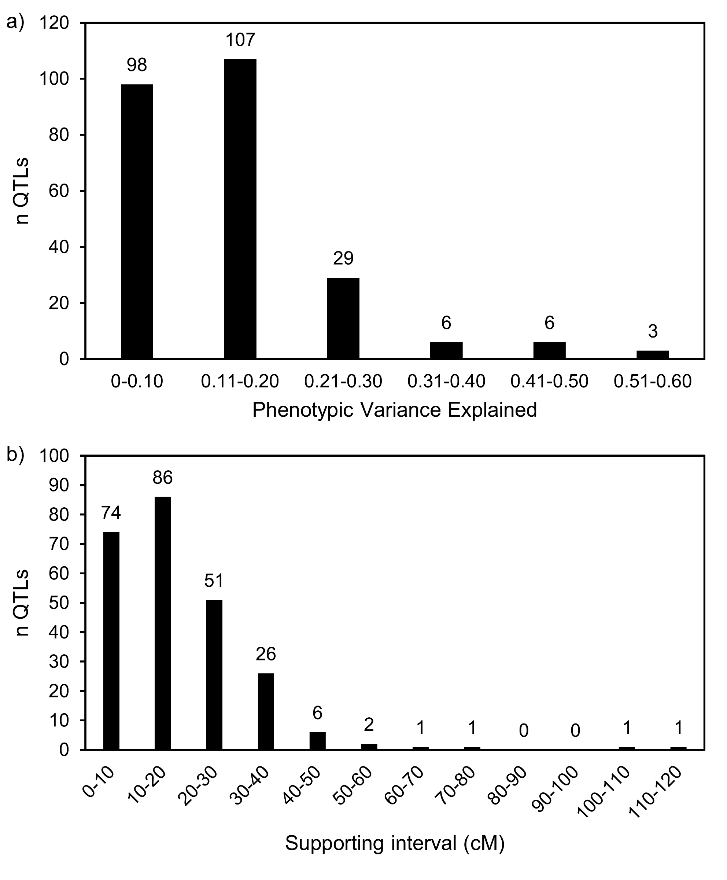


**Supplementary material 6.** Gene annotation for the most significant markers identified by association mapping. The high-confidence gene annotation from the wheat genome sequence (IWGSC RefSeq v1.0) was used.

| **Marker (Mb)** | **Trait** | **Chromosome** | **Interval (Mb)** | **Gene-ID** | **Description** |
| --- | --- | --- | --- | --- | --- |
| **wPt-3883/7734/9796 (61.8)** | Protein content | 7A | 59.8 | TraesCS7A01G097300.1 | Formate-dependent phosphoribosylglycinamide formyltransferase |
|  |  |  |  | TraesCS7A01G097400.1 | PHD finger alfin-like protein |
|  |  |  |  | TraesCS7A01G097500.1 | ATP synthase subunit alpha |
|  |  |  |  | TraesCS7A01G097600.1 | NADH-ubiquinone oxidoreductase chain 1 |
|  |  |  |  | TraesCS7A01G097700.1 | ATP synthase subunit a |
|  |  |  |  | TraesCS7A01G097800.1 | Cytochrome c oxidase subunit 3 |
|  |  |  |  | TraesCS7A01G097900.1 | Orf101b |
|  |  |  |  | TraesCS7A01G098000.1 | BnaCnng55980D protein |
|  |  |  |  | TraesCS7A01G098100.1 | Cytochrome c oxidase subunit 1 |
|  |  |  |  | TraesCS7A01G098200.1 | ATP synthase subunit 9, mitochondrial |
|  |  |  |  | TraesCS7A01G098300.1 | ATP synthase subunit alpha |
|  |  |  |  | TraesCS7A01G098400.1 | Cytochrome b559 subunit alpha |
|  |  |  |  | TraesCS7A01G098500.1 | Cytochrome c oxidase subunit 1 |
|  |  |  |  | TraesCS7A01G098600.1 | Cytochrome c oxidase subunit 2 |
|  |  |  |  | TraesCS7A01G098700.1 | NADH dehydrogenase subunit 9 |
|  |  |  |  | TraesCS7A01G098800.1 | RNA-directed RNA polymerase P1 |
|  |  |  |  | TraesCS7A01G098900.1 | NADH dehydrogenase subunit 2 |
|  |  |  |  | TraesCS7A01G099000.1 | LPS-assembly protein LptD |
|  |  |  |  | TraesCS7A01G099100.1 | Sestrin-2 |
|  |  |  |  | TraesCS7A01G099200.1 | Mitochondrial DNA orf152b |
|  |  |  |  | TraesCS7A01G099300.1 | ATPase subunit 4 |
|  |  |  |  | TraesCS7A01G099400.1 | 30S ribosomal protein S12 |
|  |  |  |  | TraesCS7A01G099500.1 | NADH-ubiquinone oxidoreductase chain 3 |
|  |  |  |  | TraesCS7A01G099600.1 | Cytochrome b559 subunit alpha |
|  |  |  |  | TraesCS7A01G099700.1 | BTB-POZ and MATH domain 2 |
|  |  |  |  | TraesCS7A01G099800.1 | Protein kinase superfamily protein |
|  |  |  |  | TraesCS7A01G099900.1 | GDSL esterase/lipase family |
|  |  |  |  | TraesCS7A01G100000.1 | Diphosphomevalonate decarboxylase |
|  |  |  |  | TraesCS7A01G100100.1 | Acetyl-coenzyme A carboxylase carboxyl transferase subunit beta |
|  |  |  |  | TraesCS7A01G100200.1 | GDSL esterase/lipase |
|  |  |  |  | TraesCS7A01G100300.1 | F-box family protein |
|  |  |  |  | TraesCS7A01G100400.1 | pre-mRNA-splicing factor CWC22-like protein (DUF3245) |
|  |  |  |  | TraesCS7A01G100500.1 | Laminin subunit beta-1 |
|  |  |  |  | TraesCS7A01G100600.1 | GDSL esterase/lipase |
|  |  |  |  | TraesCS7A01G100700.1 | Acyl-CoA synthetase family protein |
|  |  |  |  | TraesCS7A01G100800.1 | Pleckstrin homology (PH) domain-containing protein |
|  |  |  |  | TraesCS7A01G100900.1 | Maintenance of mitochondrial morphology protein 1 |
|  |  |  |  | TraesCS7A01G101000.1 | rRNA N-glycosidase |
|  |  |  |  | TraesCS7A01G101100.1 | F-box family protein |
|  |  |  |  | TraesCS7A01G101200.1 | Glycosyltransferase |
|  |  |  |  | TraesCS7A01G101300.1 | Cytochrome P450 family protein |
|  |  |  |  | TraesCS7A01G101400.1 | Calcium uniporter protein, mitochondrial |
|  |  |  |  | TraesCS7A01G101500.1 | 3'(2'),5'-bisphosphate nucleotidase 1 |
|  |  |  |  | TraesCS7A01G101600.1 | Calcium uniporter protein, mitochondrial |
|  |  |  |  | TraesCS7A01G101700.2 | Disease resistance protein RPM1 |
|  |  |  |  | TraesCS7A01G101800.1 | Xyloglucan alpha-1,6-xylosyltransferase |
|  |  |  |  | TraesCS7A01G101900.1 | Purple acid phosphatase |
|  |  |  |  | TraesCS7A01G102100.1 | disease resistance protein (TIR-NBS-LRR class) |
|  |  |  |  | TraesCS7A01G102200.1 | Pentatricopeptide repeat-containing protein |
|  |  |  |  | TraesCS7A01G102300.1 | Transmembrane protein, putative |
|  |  |  |  | TraesCS7A01G102400.1 | Nucleosome assembly protein 1-1 |
|  |  |  |  | TraesCS7A01G102500.1 | Non-canonical purine NTP pyrophosphatase |
|  |  |  |  | TraesCS7A01G102600.1 | Acetyl-coenzyme A carboxylase carboxyl transferase subunit alpha |
|  |  |  |  | TraesCS7A01G102700.1 | Cysteine synthase |
|  |  |  |  | TraesCS7A01G102800.1 | Cysteine synthase |
|  |  |  |  | TraesCS7A01G102900.1 | Histone H3 |
|  |  |  |  | TraesCS7A01G103000.1 | Protein kinase family protein |
|  |  |  |  | TraesCS7A01G103100.1 | 3'-N-debenzoyl-2'-deoxytaxol N-benzoyltransferase |
|  |  |  |  | TraesCS7A01G103200.1 | Disease resistance protein (NBS-LRR class) family |
|  |  |  |  | TraesCS7A01G103300.1 | Disease resistance protein (NBS-LRR class) family |
|  |  |  |  | TraesCS7A01G103400.1 | Disease resistance protein (NBS-LRR class) family |
|  |  |  |  | TraesCS7A01G103500.1 | Xyloglucan endotransglucosylase/hydrolase |
|  |  |  |  | TraesCS7A01G103600.1 | Xyloglucan endotransglucosylase/hydrolase |
|  |  |  |  | TraesCS7A01G103700.1 | Disease resistance protein (NBS-LRR class) family |
|  |  |  |  | TraesCS7A01G103800.1 | Histone H3 |
|  |  |  |  | TraesCS7A01G103900.1 | Histone H3 |
|  |  |  |  | TraesCS7A01G104000.1 | Eukaryotic translation initiation factor 3 subunit A |
|  |  |  |  | TraesCS7A01G104100.1 | BRISC and BRCA1-A complex member 1 |
|  |  |  |  | TraesCS7A01G104200.1 | Basic blue protein |
|  |  |  |  | TraesCS7A01G104300.1 | HXXXD-type acyl-transferase family protein |
|  |  |  |  | TraesCS7A01G104400.1 | AT4G29520-like protein |
|  |  |  | 63.5 | TraesCS7A01G104500.1 | U-box domain-containing protein |
|  |  |  | 64.6 | TraesCS7A01G106300.1 | NAC domain-containing protein |
| **wPt-2737 (199.3)** | Protein content | 7B | 184.1 | TraesCS7B01G143300.1 | Succinyl-diaminopimelate desuccinylase |
|  |  |  |  | TraesCS7B01G143400.1 | Pentatricopeptide repeat-containing protein |
|  |  |  |  | TraesCS7B01G143500.1 | Cortactin-binding protein 2 |
|  |  |  |  | TraesCS7B01G143600.3 | Leucine-rich repeat (LRR) family protein |
|  |  |  |  | TraesCS7B01G143700.1 | Uroporphyrinogen decarboxylase |
|  |  |  |  | TraesCS7B01G143800.1 | Pentatricopeptide repeat-containing protein |
|  |  |  |  | TraesCS7B01G143900.1 | NAC domain protein, |
|  |  |  |  | TraesCS7B01G144000.2 | Zinc finger CCHC domain-containing protein 24 |
|  |  |  |  | TraesCS7B01G144100.1 | RING/U-box superfamily protein |
|  |  |  |  | TraesCS7B01G144200.1 | CCAAT/enhancer-binding protein delta |
|  |  |  |  | TraesCS7B01G144300.1 | DNA-binding bromodomain-containing protein, putative |
|  |  |  |  | TraesCS7B01G144400.1 | 3-oxoacyl-reductase |
|  |  |  |  | TraesCS7B01G144500.1 | Potassium transporter |
|  |  |  |  | TraesCS7B01G144600.1 | DSBA oxidoreductase family protein |
|  |  |  |  | TraesCS7B01G144700.1 | Mitochondrial transcription termination factor family protein |
|  |  |  |  | TraesCS7B01G144800.1 | rho GTPase-activating gacO-like protein |
|  |  |  |  | TraesCS7B01G144900.1 | Squamosa promoter binding protein |
|  |  |  |  | TraesCS7B01G145000.2 | G-patch domain containing protein, expressed |
|  |  |  |  | TraesCS7B01G145100.1 | Beta-glucosidase, putative |
|  |  |  |  | TraesCS7B01G145200.1 | Lipoxygenase |
|  |  |  |  | TraesCS7B01G145300.2 | tRNA uridine 5-carboxymethylaminomethyl modification enzyme MnmG |
|  |  |  |  | TraesCS7B01G145400.1 | ETHYLENE INSENSITIVE 3-like 3 protein |
|  |  |  |  | TraesCS7B01G145500.1 | 2-oxoglutarate (2OG) and Fe(II)-dependent oxygenase superfamily protein |
|  |  |  |  | TraesCS7B01G145600.1 | IRK-interacting protein |
|  |  |  |  | TraesCS7B01G145800.1 | Basic helix-loop-helix transcription factor |
|  |  |  |  | TraesCS7B01G145900.1 | Eukaryotic translation initiation factor 2D |
|  |  |  |  | TraesCS7B01G146000.1 | tRNA/rRNA methyltransferase family protein |
|  |  |  |  | TraesCS7B01G146100.1 | Receptor-like kinase |
|  |  |  |  | TraesCS7B01G146200.1 | Lumenal PsbP-like protein |
|  |  |  |  | TraesCS7B01G146300.1 | RNA binding family protein, putative |
|  |  |  |  | TraesCS7B01G146400.1 | Zinc finger A20 and AN1 domain stress-associated protein |
|  |  |  |  | TraesCS7B01G146500.1 | Protein kinase |
|  |  |  |  | TraesCS7B01G146600.1 | 60S ribosomal protein L31 |
|  |  |  |  | TraesCS7B01G146700.1 | Nucleolar-like protein |
|  |  |  |  | TraesCS7B01G146800.1 | Leucine-rich repeat receptor-like protein kinase family |
|  |  |  |  | TraesCS7B01G146900.1 | Beta-1,2-xylosyltransferase |
|  |  |  |  | TraesCS7B01G147000.1 | Chymotrypsin inhibitor |
|  |  |  |  | TraesCS7B01G147200.1 | cysteine-rich RLK (RECEPTOR-like protein kinase) 21 |
|  |  |  |  | TraesCS7B01G147300.1 | Microtubule-associated protein RP/EB family member |
|  |  |  |  | TraesCS7B01G147400.1 | Zinc finger-like protein |
|  |  |  |  | TraesCS7B01G147500.1 | Glycerophosphodiester phosphodiesterase |
|  |  |  |  | TraesCS7B01G147600.1 | E3 ubiquitin-protein ligase |
|  |  |  |  | TraesCS7B01G147700.1 | Eukaryotic translation initiation factor 3 subunit A |
|  |  |  |  | TraesCS7B01G147800.1 | Nuclear transport factor 2 family protein, putative |
|  |  |  |  | TraesCS7B01G147900.1 | Hippocampus abundant transcript-like protein 1 |
|  |  |  |  | TraesCS7B01G148000.1 | Alanine:glyoxylate aminotransferase |
|  |  |  |  | TraesCS7B01G148100.1 | Fasciclin-like arabinogalactan protein |
|  |  |  |  | TraesCS7B01G148200.1 | Protein ENHANCED DOWNY MILDEW 2 |
|  |  |  |  | TraesCS7B01G148300.1 | WD40 domain-containing protein |
|  |  |  |  | TraesCS7B01G148400.1 | DNA double-strand break repair and VJ recombination XRCC4 |
|  |  |  |  | TraesCS7B01G148500.1 | Receptor-like protein kinase |
|  |  |  |  | TraesCS7B01G148600.1 | Protein kinase family protein |
|  |  |  |  | TraesCS7B01G148700.1 | Protein kinase family protein |
|  |  |  |  | TraesCS7B01G148800.1 | Receptor-like protein kinase |
|  |  |  |  | TraesCS7B01G148900.1 | Methionyl-tRNA formyltransferase |
|  |  |  |  | TraesCS7B01G149000.2 | Ubiquitin-associated domain-containing family protein |
|  |  |  |  | TraesCS7B01G149100.1 | Sodium/hydrogen exchanger |
|  |  |  |  | TraesCS7B01G149200.1 | Heat shock protein 90 |
|  |  |  |  | TraesCS7B01G149300.1 | Pentatricopeptide repeat-containing protein |
|  |  |  |  | TraesCS7B01G149400.1 | Nuclear factor related to kappa-B-binding protein |
|  |  |  |  | TraesCS7B01G149500.2 | Myosin heavy chain-like protein |
|  |  |  |  | TraesCS7B01G149600.1 | Chorismate mutase |
|  |  |  |  | TraesCS7B01G149700.1 | Zinc finger protein |
|  |  |  |  | TraesCS7B01G149800.1 | Mitochondrial transcription termination factor family protein |
|  |  |  |  | TraesCS7B01G149900.1 | TBC1 domain family member-like protein |
|  |  |  |  | TraesCS7B01G150000.1 | Ring finger protein, putative |
|  |  |  |  | TraesCS7B01G150100.1 | Pathogenesis-related thaumatin family protein |
|  |  |  |  | TraesCS7B01G150200.1 | Oxysterol-binding protein-like |
|  |  |  |  | TraesCS7B01G150300.1 | Methyltransferase-like protein 13 |
|  |  |  |  | TraesCS7B01G150400.1 | Pyridoxamine 5'-phosphate oxidase-related, FMN-binding |
|  |  |  |  | TraesCS7B01G150500.1 | Serrate RNA effector molecule |
|  |  |  |  | TraesCS7B01G150600.7 | Calcium-transporting ATPase |
|  |  |  |  | TraesCS7B01G150700.1 | MTD1 |
|  |  |  |  | TraesCS7B01G150800.1 | Peroxidase |
|  |  |  |  | TraesCS7B01G150900.1 | Cyclin-dependent protein kinase inhibitor SMR3 |
|  |  |  |  | TraesCS7B01G151000.1 | Serine/threonine-protein phosphatase 7 long form-like protein |
|  |  |  |  | TraesCS7B01G151100.1 | BTB/POZ domain containing protein |
|  |  |  |  | TraesCS7B01G151200.1 | RNA-binding CRS1 / YhbY (CRM) domain protein |
|  |  |  |  | TraesCS7B01G151300.1 | UDP-N-acetylglucosamine 1-carboxyvinyltransferase 1 |
|  |  |  |  | TraesCS7B01G151400.1 | BTB/POZ and MATH domain-containing protein 2 |
|  |  |  |  | TraesCS7B01G151500.1 | BTB/POZ and MATH domain-containing protein 2 |
|  |  |  |  | TraesCS7B01G151600.1 | BTB/POZ domain containing protein, expressed |
|  |  |  |  | TraesCS7B01G151700.1 | ADP-ribosylation factor, putative |
|  |  |  |  | TraesCS7B01G151800.1 | Cytochrome b-c1 complex subunit Rieske, mitochondrial |
|  |  |  |  | TraesCS7B01G151900.2 | Type II inositol 5-phosphatase, putative |
|  |  |  |  | TraesCS7B01G152000.1 | Cytochrome b561 and DOMON domain-containing protein |
|  |  |  |  | TraesCS7B01G152100.1 | Cyst nematode resistance protein-like |
|  |  |  |  | TraesCS7B01G152200.1 | Auxin-induced in root cultures protein 12 |
|  |  |  |  | TraesCS7B01G152300.1 | 60S ribosomal protein L32 |
|  |  |  |  | TraesCS7B01G152400.1 | Epidermal patterning factor-like protein 9 |
|  |  |  |  | TraesCS7B01G152500.1 | DNA topoisomerase |
|  |  |  |  | TraesCS7B01G152600.1 | Ras family |
|  |  |  |  | TraesCS7B01G152700.1 | Jacalin lectin family protein |
|  |  |  |  | TraesCS7B01G152800.1 | Basic helix-loop-helix transcription factor |
|  |  |  |  | TraesCS7B01G152900.1 | 60S ribosomal protein L32 |
|  |  |  |  | TraesCS7B01G153000.1 | Pentatricopeptide repeat-containing protein |
|  |  |  |  | TraesCS7B01G153100.1 | Peptidylprolyl isomerase |
|  |  |  |  | TraesCS7B01G153200.1 | VWA domain containing protein |
|  |  |  |  | TraesCS7B01G153300.1 | Glutathione S-transferase T3 |
|  |  |  |  | TraesCS7B01G153400.1 | Nudix hydrolase |
|  |  |  |  | TraesCS7B01G153600.1 | Glucan endo-1,3-beta-glucosidase-like protein |
|  |  |  |  | TraesCS7B01G153700.1 | myb-like protein X |
|  |  |  |  | TraesCS7B01G153800.1 | Choline kinase |
|  |  |  |  | TraesCS7B01G153900.1 | UDP-glucuronate 4-epimerase 4 |
|  |  |  |  | TraesCS7B01G154000.1 | RING/U-box superfamily protein |
|  |  |  |  | TraesCS7B01G154100.1 | RPM1-interacting protein 4 |
|  |  |  |  | TraesCS7B01G154200.1 | Sterile alpha motif (SAM) domain-containing protein |
|  |  |  |  | TraesCS7B01G154300.1 | FHA domain containing protein, expressed |
|  |  |  |  | TraesCS7B01G154400.1 | UBX domain-containing protein |
|  |  |  |  | TraesCS7B01G154500.1 | Werner Syndrome-like exonuclease |
|  |  |  |  | TraesCS7B01G154600.1 | RING/U-box superfamily protein |
|  |  |  |  | TraesCS7B01G154700.1 | Ubiquitinyl hydrolase 1 |
|  |  |  |  | TraesCS7B01G154800.1 | Ubiquitinyl hydrolase 1 |
|  |  |  |  | TraesCS7B01G154900.1 | Ribosomal protein L2 |
|  |  |  |  | TraesCS7B01G155100.1 | Serine/threonine-protein kinase flr-4 |
|  |  |  |  | TraesCS7B01G155200.1 | Werner Syndrome-like exonuclease |
|  |  |  |  | TraesCS7B01G155300.1 | Serine/threonine-protein kinase flr-4 |
|  |  |  |  | TraesCS7B01G155400.1 | Myb/SANT-like DNA-binding domain protein |
|  |  |  | 214.0 | TraesCS7B01G155500.1 | Ubiquitinyl hydrolase 1 |
| **wPt-1140 (601.9)** | Test weight | 2B | 599.8 | TraesCS2B01G418600.1 | Cytochrome P450 |
|  |  |  |  | TraesCS2B01G418700.1 | Cytochrome P450 family protein |
|  |  |  |  | TraesCS2B01G418800.1 | Myb/SANT-like DNA-binding domain protein |
|  |  |  |  | TraesCS2B01G418900.1 | IQ domain-containing protein |
|  |  |  |  | TraesCS2B01G419000.1 | S-adenosyl-L-methionine-dependent methyltransferases superfamily protein |
|  |  |  |  | TraesCS2B01G419100.1 | Rhomboid-like protein |
|  |  |  |  | TraesCS2B01G419200.1 | Homeobox-leucine zipper protein |
|  |  |  |  | TraesCS2B01G419300.1 | Replication factor C subunit |
|  |  |  |  | TraesCS2B01G419400.1 | E3 ubiquitin-protein ligase |
|  |  |  |  | TraesCS2B01G419500.1 | DNA topoisomerase 4 subunit B (DUF810) |
|  |  |  |  | TraesCS2B01G419600.1 | Heat Stress Transcription Factor family protein |
|  |  |  |  | TraesCS2B01G419700.1 | Ribosomal protein L19 |
|  |  |  |  | TraesCS2B01G419800.1 | RING finger protein |
|  |  |  |  | TraesCS2B01G419900.1 | 3-hexulose-6-phosphate isomerase |
|  |  |  |  | TraesCS2B01G420000.1 | Bax inhibitor 1 |
|  |  |  |  | TraesCS2B01G420100.1 | WD-repeat protein, putative |
|  |  |  |  | TraesCS2B01G420200.1 | DNA topoisomerase |
|  |  |  |  | TraesCS2B01G420300.1 | Actin-depolymerizing factor 5 |
|  |  |  |  | TraesCS2B01G420400.1 | Dof zinc finger protein |
|  |  |  |  | TraesCS2B01G420500.1 | ORMDL family protein-like |
|  |  |  |  | TraesCS2B01G420600.1 | Aluminum-activated malate transporter-like |
|  |  |  |  | TraesCS2B01G420700.1 | Mitochondrial import inner membrane translocase subunit tim23 |
|  |  |  |  | TraesCS2B01G420800.1 | Glutathione S-transferase |
|  |  |  | 603.9 | TraesCS2B01G420900.1 | two-component response regulator |
| **wPt-6204 (7.9)** | Test weight | 3A | 5.6 | TraesCS3A01G005200.1 | Glutamate--cysteine ligase |
|  |  |  |  | TraesCS3A01G005300.1 | B3 domain-containing protein |
|  |  |  |  | TraesCS3A01G005400.1 | defensin-like protein |
|  |  |  |  | TraesCS3A01G005500.1 | MLO-like protein |
|  |  |  |  | TraesCS3A01G005600.1 | Pectinesterase inhibitor |
|  |  |  |  | TraesCS3A01G005700.1 | Pectinesterase inhibitor |
|  |  |  |  | TraesCS3A01G005800.1 | Pectinesterase inhibitor |
|  |  |  |  | TraesCS3A01G005900.1 | Peptidyl-prolyl cis-trans isomerase |
|  |  |  |  | TraesCS3A01G006000.1 | Pectinesterase inhibitor |
|  |  |  |  | TraesCS3A01G006100.1 | Protein decapping 5 |
|  |  |  |  | TraesCS3A01G006300.1 | basic helix-loop-helix (bHLH) DNA-binding superfamily protein |
|  |  |  |  | TraesCS3A01G006400.1 | Cell division cycle protein 123 like |
|  |  |  |  | TraesCS3A01G006600.1 | FLOWERING LOCUS T/ TERMINAL FLOWER 1-like protein |
|  |  |  |  | TraesCS3A01G006700.1 | Protein kinase family protein |
|  |  |  |  | TraesCS3A01G006800.1 | Wall-associated receptor kinase 2 |
|  |  |  |  | TraesCS3A01G006900.1 | Protein kinase family protein |
|  |  |  |  | TraesCS3A01G007000.1 | Transmembrane protein, putative |
|  |  |  |  | TraesCS3A01G007100.1 | WAT1-related protein |
|  |  |  |  | TraesCS3A01G007200.1 | Receptor-kinase, putative |
|  |  |  |  | TraesCS3A01G007300.1 | 50S ribosomal protein L6 |
|  |  |  |  | TraesCS3A01G007400.1 | Disease resistance protein (TIR-NBS-LRR class) |
|  |  |  |  | TraesCS3A01G007500.1 | Misshapen-like kinase 1 |
|  |  |  |  | TraesCS3A01G007600.1 | Transcription elongation factor Spt5 |
|  |  |  |  | TraesCS3A01G007700.1 | CheY-like two-component responsive regulator family protein |
|  |  |  |  | TraesCS3A01G007800.1 | GDSL esterase/lipase |
|  |  |  |  | TraesCS3A01G007900.1 | Monoterpene synthase |
|  |  |  |  | TraesCS3A01G008000.1 | Disease resistance protein (NBS-LRR class) family |
|  |  |  |  | TraesCS3A01G008100.1 | Disease resistance protein RPM1 |
|  |  |  |  | TraesCS3A01G008200.1 | Disease resistance protein (NBS-LRR class) family |
|  |  |  |  | TraesCS3A01G008300.1 | Disease resistance protein (TIR-NBS-LRR class) family |
|  |  |  |  | TraesCS3A01G008400.1 | Ubiquitin-like-specific protease 1D |
|  |  |  |  | TraesCS3A01G008500.1 | F6A14.6 protein |
|  |  |  |  | TraesCS3A01G008600.1 | disease resistance protein (TIR-NBS-LRR class) |
|  |  |  |  | TraesCS3A01G008700.1 | Aquaporin-1 |
|  |  |  |  | TraesCS3A01G008800.1 | Kinase family protein |
|  |  |  |  | TraesCS3A01G008900.1 | Receptor kinase-like protein |
|  |  |  |  | TraesCS3A01G009000.1 | Receptor kinase-like protein |
|  |  |  |  | TraesCS3A01G009100.1 | Kinase family protein |
|  |  |  |  | TraesCS3A01G009200.1 | NBS-LRR-like resistance protein |
|  |  |  |  | TraesCS3A01G009300.1 | Disease resistance protein (TIR-NBS-LRR class) family |
|  |  |  |  | TraesCS3A01G009400.2 | RNA polymerase II-associated factor 1 |
|  |  |  |  | TraesCS3A01G009500.1 | Invertase inhibitor |
|  |  |  |  | TraesCS3A01G009600.1 | U-box domain-containing protein |
|  |  |  |  | TraesCS3A01G009700.1 | BTB/POZ domain containing protein, expressed |
|  |  |  |  | TraesCS3A01G009800.1 | Receptor-like protein kinase |
|  |  |  |  | TraesCS3A01G009900.1 | Nbs-lrr resistance protein, putative |
|  |  |  |  | TraesCS3A01G010000.1 | TPX2 (Targeting protein for Xklp2) family protein |
|  |  |  |  | TraesCS3A01G010100.1 | Dephospho-CoA kinase, putative, expressed |
|  |  |  |  | TraesCS3A01G010200.1 | Invertase inhibitor |
|  |  |  |  | TraesCS3A01G010300.1 | Glycosyltransferase |
|  |  |  |  | TraesCS3A01G010400.1 | Agenet domain containing protein |
|  |  |  |  | TraesCS3A01G010500.1 | Thyrotropin-releasing hormone-degrading ectoenzyme |
|  |  |  |  | TraesCS3A01G010600.1 | PII-type proteinase |
|  |  |  |  | TraesCS3A01G010700.1 | poly(A) binding protein 8 |
|  |  |  |  | TraesCS3A01G010800.1 | Vacuolar protein sorting-associated protein 4 |
|  |  |  |  | TraesCS3A01G010900.1 | Flavin-containing monooxygenase |
|  |  |  |  | TraesCS3A01G011000.1 | Leucine--tRNA ligase |
|  |  |  |  | TraesCS3A01G011100.1 | Expansin protein |
|  |  |  |  | TraesCS3A01G011200.1 | Expansin protein |
|  |  |  |  | TraesCS3A01G011300.1 | S-adenosyl-L-methionine-dependent methyltransferases superfamily protein |
|  |  |  |  | TraesCS3A01G011400.1 | Major Facilitator Superfamily with SPX (SYG1/Pho81/XPR1) domain-containing protein |
|  |  |  |  | TraesCS3A01G011500.1 | Fasciclin-like arabinogalactan, putative, expressed |
|  |  |  |  | TraesCS3A01G011600.1 | Myozenin-2 |
|  |  |  |  | TraesCS3A01G011800.1 | Translation initiation factor IF-2 |
|  |  |  |  | TraesCS3A01G011900.1 | beta glucosidase 18 |
|  |  |  |  | TraesCS3A01G012000.1 | Subtilisin-like protease 1 |
|  |  |  |  | TraesCS3A01G012200.1 | Reticulon family protein |
|  |  |  |  | TraesCS3A01G012300.1 | Tetratricopeptide repeat protein, tpr, putative |
|  |  |  |  | TraesCS3A01G012400.1 | Germin-like protein |
|  |  |  |  | TraesCS3A01G012500.1 | Germin-like protein |
|  |  |  |  | TraesCS3A01G012600.1 | Germin-like protein |
|  |  |  |  | TraesCS3A01G012700.1 | Coatomer subunit beta'-1 |
|  |  |  |  | TraesCS3A01G012800.2 | Pentatricopeptide repeat-containing protein |
|  |  |  |  | TraesCS3A01G012900.1 | Receptor kinase |
|  |  |  |  | TraesCS3A01G013000.1 | Receptor kinase |
|  |  |  |  | TraesCS3A01G013100.1 | Flowering Locus T-like protein, putative |
|  |  |  |  | TraesCS3A01G013200.1 | ERECTA-like 1 |
|  |  |  |  | TraesCS3A01G013300.1 | Protein disulfide isomerase-like 1-1 |
|  |  |  |  | TraesCS3A01G013400.1 | Receptor kinase |
|  |  |  |  | TraesCS3A01G013500.1 | Receptor kinase |
|  |  |  |  | TraesCS3A01G013600.1 | O-acyltransferase WSD1 |
|  |  |  |  | TraesCS3A01G013700.2 | O-acyltransferase WSD1 |
|  |  |  |  | TraesCS3A01G013800.1 | Fatty acyl-CoA reductase |
|  |  |  |  | TraesCS3A01G013900.1 | NBS-LRR-like resistance protein |
|  |  |  |  | TraesCS3A01G014000.1 | Fatty acyl-CoA reductase |
|  |  |  |  | TraesCS3A01G014100.1 | Fatty acyl-CoA reductase |
|  |  |  |  | TraesCS3A01G014200.1 | NBS-LRR-like resistance protein |
|  |  |  |  | TraesCS3A01G014300.1 | DNA topoisomerase |
|  |  |  |  | TraesCS3A01G014400.1 | DNA topoisomerase |
|  |  |  |  | TraesCS3A01G014500.1 | Disease resistance protein RPM1 |
|  |  |  |  | TraesCS3A01G014600.1 | Disease resistance protein |
|  |  |  |  | TraesCS3A01G014700.1 | Receptor-like protein kinase |
|  |  |  |  | TraesCS3A01G014800.1 | Fatty acyl-CoA reductase |
|  |  |  | 10.2 | TraesCS3A01G014900.1 | Fatty acyl-CoA reductase |
| **wPt-3729 (735.3)** | Yellow colour | 4A | 732.7 | TraesCS4A01G472400.1 | WAT1-related protein |
|  |  |  |  | TraesCS4A01G472500.1 | Ferredoxin--NADP reductase |
|  |  |  |  | TraesCS4A01G472600.1 | SKP1-like protein |
|  |  |  |  | TraesCS4A01G472700.1 | B3 domain-containing protein |
|  |  |  |  | TraesCS4A01G472800.1 | Aspartic proteinase nepenthesin-1 |
|  |  |  |  | TraesCS4A01G472900.1 | Aspartate carbamoyltransferase |
|  |  |  |  | TraesCS4A01G473000.1 | Alpha-ketoglutarate-dependent sulfate ester dioxygenase |
|  |  |  |  | TraesCS4A01G473100.1 | cyclin delta-3 |
|  |  |  |  | TraesCS4A01G473200.1 | Chaperone protein DnaK |
|  |  |  |  | TraesCS4A01G473300.1 | Leucine-rich receptor-like protein kinase family protein |
|  |  |  |  | TraesCS4A01G473400.1 | Protein CWC15 like |
|  |  |  |  | TraesCS4A01G473500.1 | Hydroxycinnamoyl-CoA shikimate/quinate hydroxycinnamoyltransferase |
|  |  |  |  | TraesCS4A01G473600.1 | keratin-associated protein (DUF819) |
|  |  |  |  | TraesCS4A01G473700.1 | Potassium channel toxin alpha-KTx 3.18 |
|  |  |  |  | TraesCS4A01G473800.1 | F-box protein |
|  |  |  |  | TraesCS4A01G473900.1 | F-box protein |
|  |  |  |  | TraesCS4A01G474000.2 | Grain length protein |
|  |  |  |  | TraesCS4A01G474100.2 | MYB-related transcription factor |
|  |  |  |  | TraesCS4A01G474200.1 | cDNA clone:J023132J12, full insert sequence |
|  |  |  |  | TraesCS4A01G474300.1 | Site-determining protein |
|  |  |  |  | TraesCS4A01G474400.1 | F-box protein |
|  |  |  |  | TraesCS4A01G474500.1 | Non-canonical purine NTP pyrophosphatase |
|  |  |  |  | TraesCS4A01G474600.1 | F-box protein |
|  |  |  |  | TraesCS4A01G474700.1 | Disease resistance protein |
|  |  |  |  | TraesCS4A01G474800.2 | Receptor protein kinase, putative |
|  |  |  |  | TraesCS4A01G474900.1 | Disease resistance protein (TIR-NBS-LRR class) family |
|  |  |  |  | TraesCS4A01G475000.1 | Coatomer, beta' subunit |
|  |  |  |  | TraesCS4A01G475100.1 | Disease resistance protein (NBS-LRR class) family |
|  |  |  |  | TraesCS4A01G475200.1 | Disease resistance protein (CC-NBS-LRR class) family |
|  |  |  |  | TraesCS4A01G475300.1 | NSP-interacting kinase 2 |
|  |  |  |  | TraesCS4A01G475400.1 | Rp1-like protein |
|  |  |  |  | TraesCS4A01G475500.1 | molybdopterin biosynthesis MoaE family protein |
|  |  |  |  | TraesCS4A01G475600.1 | Multifunctional CCA protein |
|  |  |  |  | TraesCS4A01G475700.1 | Rp1-like protein |
|  |  |  |  | TraesCS4A01G475800.1 | Receptor kinase |
|  |  |  |  | TraesCS4A01G475900.2 | NBS-LRR-like resistance protein |
|  |  |  |  | TraesCS4A01G476000.1 | receptor kinase 1 |
|  |  |  |  | TraesCS4A01G476100.1 | Rp1-like protein |
|  |  |  |  | TraesCS4A01G476200.1 | NBS-LRR-like resistance protein |
|  |  |  |  | TraesCS4A01G476300.1 | Rp1-like protein |
|  |  |  |  | TraesCS4A01G476400.1 | disease resistance family protein / LRR family protein |
|  |  |  |  | TraesCS4A01G476500.1 | Rp1-like protein |
|  |  |  |  | TraesCS4A01G476600.1 | Rp1-like protein |
|  |  |  |  | TraesCS4A01G476700.1 | Rp1-like protein |
|  |  |  |  | TraesCS4A01G476800.1 | Cytochrome P450 family protein, expressed |
|  |  |  |  | TraesCS4A01G476900.1 | Cytochrome P450 family protein, expressed |
|  |  |  |  | TraesCS4A01G477000.1 | Multifunctional CCA protein |
|  |  |  |  | TraesCS4A01G477100.1 | GRF zinc finger family protein |
|  |  |  |  | TraesCS4A01G477200.1 | Serine/threonine-protein phosphatase 7 long form-like protein |
|  |  |  |  | TraesCS4A01G477300.1 | Disease resistance protein (NBS-LRR class) family |
|  |  |  |  | TraesCS4A01G477400.1 | Rp1-like protein |
|  |  |  |  | TraesCS4A01G477500.1 | Phenylalanine--tRNA ligase alpha subunit |
|  |  |  |  | TraesCS4A01G477600.1 | Rp1-like protein |
|  |  |  |  | TraesCS4A01G477700.1 | Disease resistance protein (NBS-LRR class) family |
|  |  |  |  | TraesCS4A01G477800.1 | Rp1-like protein |
|  |  |  |  | TraesCS4A01G477900.1 | NADH-ubiquinone oxidoreductase chain 2 |
|  |  |  |  | TraesCS4A01G478000.1 | Rp1-like protein |
|  |  |  |  | TraesCS4A01G478100.1 | Rp1-like protein |
|  |  |  |  | TraesCS4A01G478200.1 | NBS-LRR-like resistance protein |
|  |  |  |  | TraesCS4A01G478300.1 | Rp1-like protein |
|  |  |  |  | TraesCS4A01G478400.1 | Rp1-like protein |
|  |  |  |  | TraesCS4A01G478500.1 | Phenylalanine--tRNA ligase alpha subunit |
|  |  |  |  | TraesCS4A01G478600.1 | Rp1-like protein |
|  |  |  |  | TraesCS4A01G478700.1 | Receptor kinase |
|  |  |  |  | TraesCS4A01G478800.1 | Rp1-like protein |
|  |  |  |  | TraesCS4A01G478900.1 | Rp1-like protein |
|  |  |  |  | TraesCS4A01G479100.1 | Disease resistance protein (NBS-LRR class) family |
|  |  |  |  | TraesCS4A01G479200.1 | Leucine-rich repeat receptor-like protein kinase family protein |
|  |  |  |  | TraesCS4A01G479300.1 | Receptor kinase |
|  |  |  |  | TraesCS4A01G479400.1 | Rp1-like protein |
|  |  |  |  | TraesCS4A01G479500.1 | Multifunctional CCA protein |
|  |  |  |  | TraesCS4A01G479600.1 | Histidine-containing phosphotransfer protein |
|  |  |  |  | TraesCS4A01G479700.1 | Rp1-like protein |
|  |  |  |  | TraesCS4A01G479800.1 | receptor kinase 1 |
|  |  |  |  | TraesCS4A01G479900.1 | NBS-LRR-like resistance protein |
|  |  |  |  | TraesCS4A01G480000.1 | Rp1-like protein |
|  |  |  |  | TraesCS4A01G480100.1 | Rp1-like protein |
|  |  |  |  | TraesCS4A01G480200.1 | Rp1-like protein |
|  |  |  |  | TraesCS4A01G480300.1 | Rp1-like protein |
|  |  |  |  | TraesCS4A01G480400.1 | Cytochrome P450 family protein, expressed |
|  |  |  |  | TraesCS4A01G480500.1 | hydroxysteroid dehydrogenase 3 |
|  |  |  |  | TraesCS4A01G480600.1 | Histidine-containing phosphotransfer protein |
|  |  |  |  | TraesCS4A01G480700.2 | Histidine-containing phosphotransfer protein |
|  |  |  |  | TraesCS4A01G480800.1 | Histidine-containing phosphotransfer protein |
|  |  |  |  | TraesCS4A01G480900.1 | Mitochondrial pyruvate carrier |
|  |  |  |  | TraesCS4A01G481000.1 | Glycosyltransferase |
|  |  |  |  | TraesCS4A01G481100.1 | Cone cGMP-specific 3',5'-cyclic phosphodiesterase subunit alpha' |
|  |  |  |  | TraesCS4A01G481200.1 | protein kinase family protein |
|  |  |  |  | TraesCS4A01G481300.1 | receptor kinase 1 |
|  |  |  |  | TraesCS4A01G481400.1 | Protein transport protein SEC16B-like protein |
|  |  |  |  | TraesCS4A01G481500.1 | F-box family protein |
|  |  |  |  | TraesCS4A01G481600.1 | receptor kinase 1 |
|  |  |  |  | TraesCS4A01G481700.1 | Arginine N-methyltransferase family protein |
|  |  |  |  | TraesCS4A01G481800.3 | Acetyltransferase component of pyruvate dehydrogenase complex |
|  |  |  |  | TraesCS4A01G481900.1 | Disease resistance protein (TIR-NBS-LRR class) family |
|  |  |  |  | TraesCS4A01G482000.1 | Scarecrow transcription factor family protein |
|  |  |  |  | TraesCS4A01G482100.1 | 50S ribosomal protein L9 |
|  |  |  |  | TraesCS4A01G482300.1 | Tetratricopeptide repeat protein 7A |
|  |  |  |  | TraesCS4A01G482400.1 | Receptor-like protein kinase |
|  |  |  |  | TraesCS4A01G482500.1 | Receptor-like kinase |
|  |  |  | 737.8 | TraesCS4A01G482600.1 | Receptor-like kinase |
| **wPt-7653 (60.3)** | Gluten strength | 7B | 57.6 | TraesCS7B01G054400.1 | Glutathione peroxidase |
|  |  |  |  | TraesCS7B01G054500.1 | Receptor-kinase, putative |
|  |  |  |  | TraesCS7B01G054600.1 | Receptor kinase-like protein |
|  |  |  |  | TraesCS7B01G054700.1 | Tetratricopeptide repeat (TPR)-like superfamily protein |
|  |  |  |  | TraesCS7B01G054800.1 | Alpha subunit of RNA polymerase |
|  |  |  |  | TraesCS7B01G054900.1 | Carboxypeptidase |
|  |  |  |  | TraesCS7B01G055000.1 | HAUS augmin-like complex subunit 3 |
|  |  |  |  | TraesCS7B01G055100.1 | Nucleoporin NUP188 |
|  |  |  |  | TraesCS7B01G055200.2 | Thiosulfate sulfurtransferase GlpE |
|  |  |  |  | TraesCS7B01G055300.1 | ATP-dependent DNA helicase |
|  |  |  |  | TraesCS7B01G055400.1 | Major facilitator superfamily protein |
|  |  |  |  | TraesCS7B01G055500.1 | DNA topoisomerase |
|  |  |  |  | TraesCS7B01G055600.1 | Origin recognition complex subunit 1 |
|  |  |  |  | TraesCS7B01G055700.1 | Methyltransferase |
|  |  |  |  | TraesCS7B01G055800.2 | Serine/threonine-protein kinase |
|  |  |  |  | TraesCS7B01G055900.1 | Serine/threonine-protein kinase |
|  |  |  |  | TraesCS7B01G056000.1 | Glycosyltransferase |
|  |  |  |  | TraesCS7B01G056100.1 | Pathogenesis-related protein 1 |
|  |  |  |  | TraesCS7B01G056200.1 | UDP-glucuronate 4-epimerase 4 |
|  |  |  |  | TraesCS7B01G056300.1 | NAC domain protein |
|  |  |  |  | TraesCS7B01G056400.1 | RING finger protein |
|  |  |  |  | TraesCS7B01G056500.1 | E3 ubiquitin-protein ligase SINA-like 10 |
|  |  |  |  | TraesCS7B01G056600.2 | Arginine/serine-rich splicing factor, putative |
|  |  |  |  | TraesCS7B01G056700.1 | Cyclic nucleotide-gated channel |
|  |  |  |  | TraesCS7B01G056800.1 | TIR-NBS-LRR class disease resistance protein |
|  |  |  |  | TraesCS7B01G056900.1 | Disease resistance protein (NBS-LRR class) family |
|  |  |  |  | TraesCS7B01G057000.1 | winged-helix DNA-binding transcription factor family protein |
|  |  |  |  | TraesCS7B01G057100.1 | UAA transporter |
|  |  |  |  | TraesCS7B01G057200.1 | Glutamate receptor |
|  |  |  |  | TraesCS7B01G057300.1 | 7-cyano-7-deazaguanine synthase |
|  |  |  |  | TraesCS7B01G057400.1 | Glutamate receptor |
|  |  |  |  | TraesCS7B01G057500.1 | Glutathione S-transferase |
|  |  |  |  | TraesCS7B01G057600.1 | Glutathione S-transferase T3 |
|  |  |  |  | TraesCS7B01G057700.1 | Glutamate receptor |
|  |  |  |  | TraesCS7B01G057800.1 | Glutamate receptor |
|  |  |  |  | TraesCS7B01G057900.1 | Glutamate receptor |
|  |  |  |  | TraesCS7B01G058000.1 | Glutathione S-transferase |
|  |  |  |  | TraesCS7B01G058100.1 | Glutamate receptor |
|  |  |  |  | TraesCS7B01G058200.1 | Glutamate receptor |
|  |  |  |  | TraesCS7B01G058300.1 | Glutamate receptor |
|  |  |  |  | TraesCS7B01G058400.1 | Glutamate receptor |
|  |  |  |  | TraesCS7B01G058500.1 | Mannitol transporter, putative, expressed |
|  |  |  |  | TraesCS7B01G058600.1 | Gibberellin receptor GID1A |
|  |  |  |  | TraesCS7B01G058700.1 | Lysine-specific demethylase 5A |
|  |  |  |  | TraesCS7B01G058800.1 | Glutamate receptor |
|  |  |  |  | TraesCS7B01G058900.1 | zinc ion binding protein |
|  |  |  |  | TraesCS7B01G059000.1 | Protein kinase |
|  |  |  |  | TraesCS7B01G059100.1 | Cytochrome P450 |
|  |  |  |  | TraesCS7B01G059200.1 | Cytochrome P450 |
|  |  |  |  | TraesCS7B01G059300.1 | Kinase |
|  |  |  |  | TraesCS7B01G059400.1 | Adenine nucleotide alpha hydrolases-domain containing protein kinase |
|  |  |  | 62.3 | TraesCS7B01G059500.1 | UDP-glycosyltransferase |
